# Persistent Nonlinear Phase-Locking and Nonmonotonic Energy Dissipation in Micromechanical Resonators

**DOI:** 10.1103/physrevx.12.041025

**Published:** 2022

**Authors:** Mingkang Wang, Diego J. Perez-Morelo, Daniel Lopez, Vladimir A. Aksyuk

**Affiliations:** 1 Microsystems and Nanotechnology Division, National Institute of Standards and Technology, Gaithersburg, Maryland 20899, USA; 2 Institute for Research in Electronics and Applied Physics, University of Maryland, College Park, Maryland 20742, USA; 3 Materials Research Institute, The Pennsylvania State University, University Park, Pennsylvania 16802, USA

**Keywords:** Mechanics, Nonlinear Dynamics

## Abstract

Many nonlinear systems are described by eigenmodes with amplitude-dependent frequencies, interacting strongly whenever the frequencies become commensurate at internal resonances. Fast energy exchange via the resonances holds the key to rich dynamical behavior, such as time-varying relaxation rates and signatures of nonergodicity in thermal equilibrium, revealed in the recent experimental and theoretical studies of micro- and nanomechanical resonators. However, a universal yet intuitive physical description for these diverse and sometimes contradictory experimental observations remains elusive. Here we experimentally reveal persistent nonlinear phase-locked states occurring at internal resonances and demonstrate that they are essential for understanding the transient dynamics of nonlinear systems with coupled eigenmodes. The measured dynamics of a fully observable micromechanical resonator system are quantitatively described by the lower-frequency mode entering, maintaining, and exiting a persistent phase-locked period-tripling state generated by the nonlinear driving force exerted by the higher-frequency mode. This model describes the observed phase-locked coherence times, the direction and magnitude of the energy exchange, and the resulting nonmonotonic mode energy evolution. Depending on the initial relative phase, the system selects distinct relaxation pathways, either entering or bypassing the locked state. The described persistent phase locking is not limited to particular frequency fractions or types of nonlinearities and may advance nonlinear resonator systems engineering across physical domains, including photonics as well as nanomechanics.

## I. INTRODUCTION

Physical systems are never fully isolated from their environment, leading to unavoidable energy exchange. Understanding the origins and effects of energy dissipation has been fundamental for the development of science and engineering fields such as quantum information [1,2], cosmology [3], acoustics [4], timing [5], and sensing [6]. The linearized description of such open systems’ dynamics, a bedrock of modern physics, leads to damped noninteracting eigenmodes, where damping and stochastic Langevin forces result from averaging over weak interactions with the many degrees of freedom of the external “thermal bath” [7]. While the exact solutions for nonlinear systems are generally hard to analytically obtain, their dynamics can often be studied by treating the nonlinearities as perturbations to the linearized system, retaining the eigenmodes and eigenfrequencies, but allowing higher harmonics, amplitude-dependent eigenfrequencies, and time-variable energy flows between eigenmodes. In low-loss nonlinear systems, these result in rich and complex dynamical behavior both in [8–10] and out of thermal equilibrium [11–13].

In recent years, micro- and nanomechanical resonators have emerged as an ideal experimental platform to study nonlinear phenomena due to the multiple accessible modes with high quality factors and controllable nonlinearities [9,14–19]. The individual mode nonlinearities and intermodal couplings allow the resonant frequencies to vary in time, following the changing amplitudes. Furthermore, the systems can dynamically tune in and out of the internal resonances [20–22] defined as resonances between system eigenmodes, wherein commensurate eigenfrequencies enable resonantly enhanced energy exchanges between two or more coupled modes [11]. Recent experiments involving nonlinear resonators made of carbon nanotubes (CNTs), graphene membranes, and nano- and micromechanical structures have demonstrated interesting and unexpected dynamical behaviors both during thermalization and in thermal equilibrium. Long-time-scale energy fluctuations were observed in the thermalized state of CNTs, indicative of nonergodic behavior [10]. Multilayer-graphene-membrane resonators have shown abrupt transitions between two different energy-relaxation regimes, with the mode-coupled state characterized by a faster relaxation rate for the observed vibrational mode [12]. In contrast, the lower-frequency vibrational mode of a nonlinear two-mode micromechanical resonator exhibited a slow, near-zero decay rate in the mode-coupled state [11]. These observations offer intriguing glimpses of nontrivial nonlinear dynamics, begging for more intuitive and universal physical descriptions of the underlying mechanisms. However, experimental progress has been hindered so far by the inability of the current experimental platforms to fully isolate and simultaneously observe all the interacting modes and to selectively prepare them in arbitrary initial excited states.

Here, we fully observe the thermalization transient dynamics of a prototypical nonlinear two-mode coupled system from an arbitrarily prepared excited state and develop a simple yet accurate quantitative model describing it. We discover that during the free-ringdown relaxation the system can either enter or bypass a persistent phase-locked state at the 1∶3 internal resonance. For the given starting energies, the outcome is determined solely by the initial relative phase between the modes. The phase-locked state arising during the free ringdown can last up to approximately 2.5× longer than the longest thermalization time in the system and is characterized by continuous energy transfer from a higher-loss-rate mode to a lower one, decreasing the overall system dissipation and making it a nonlinear function of the system energy.

The system transient dynamics can be quantitatively understood from the perspective of the lower-frequency mode experiencing a time-dependent modulation force from the higher-frequency mode [13], which creates stable degenerate period-tripling states (PTSs) [23–25]. While the PTS persists, the energy flows between the modes, maintaining the exact 1∶3 relationship between their changing, amplitude-dependent frequencies. As a result, the lower-frequency mode experiences a negative dissipation rate exhibiting a striking nonmonotonic energy evolution.

## II. ENERGY- AND PHASE-DEPENDENT RELAXATION PATHWAYS FOR COUPLED NONLINEAR RESONATORS

**FIG. 1.**
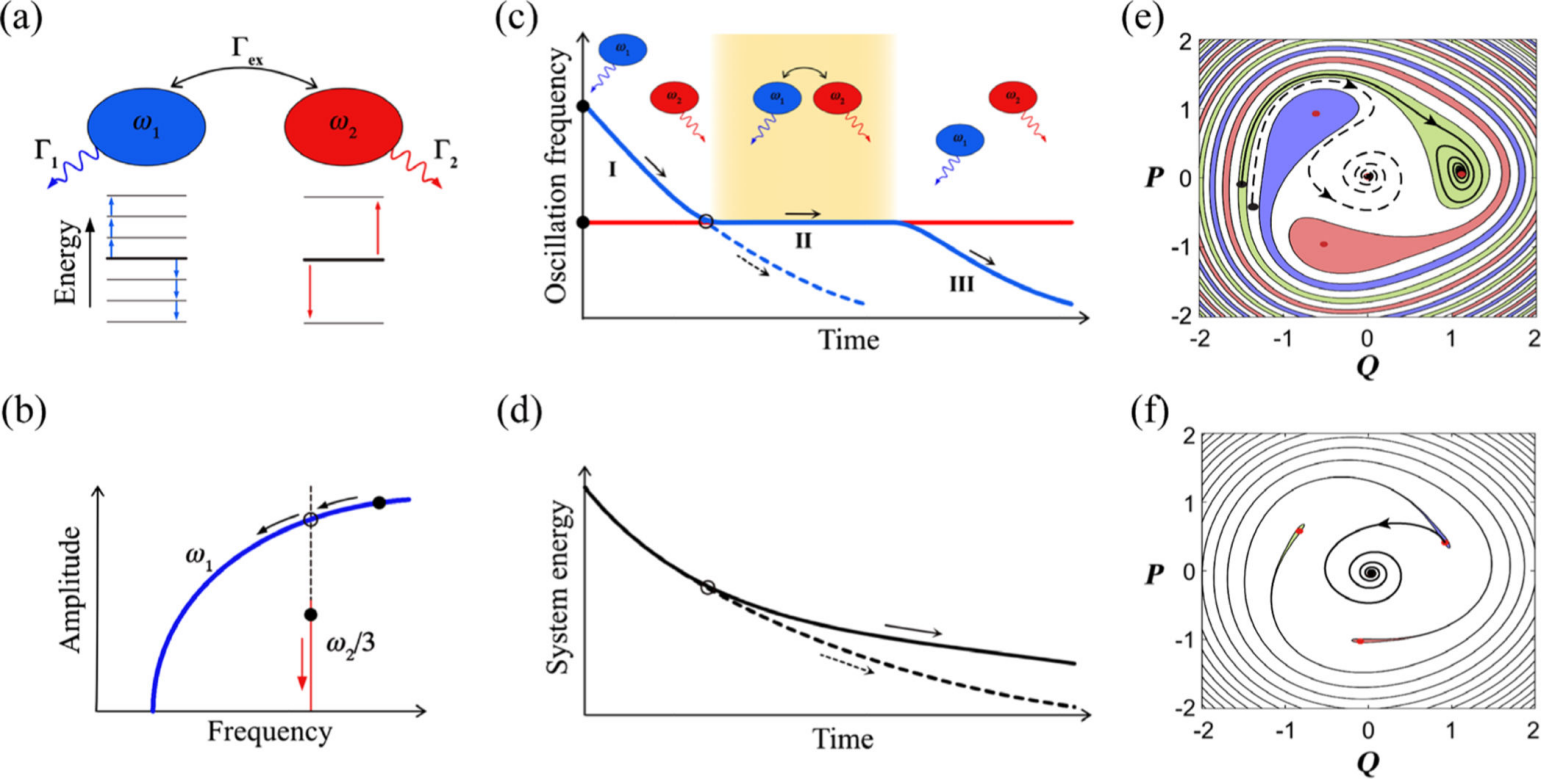
Energy exchange and relaxation paths of a system consisting of two nonlinear coupled oscillators. (a) Schematic diagram showing the energy exchanged between two nonlinear coupled oscillators with commensurate frequencies (here $\omega_{1}:\omega_{2}=1:3$) and with the thermal bath. (b) Nonlinearity of mode 1 allows tuning of its oscillation frequency $\omega_{1}$ above, at, or below the internal resonance $\omega_{2}$/3 as a function of the mode 1 amplitude, as shown in a schematic of the individual modes’ spectral responses. (c) Oscillation frequency of mode 1 (blue) and mode 2 (red) during ringdown. The oscillation frequency of mode 1 decreases during ringdown due to positive Duffing nonlinearity. At the internal resonance (black circle), mode 1 enters (solid line) or bypasses (dashed line) a phase-locked state where resonantly enhanced energy exchange with mode 2 occurs. (d) Combined energy of the two-oscillator system. The system of coupled oscillators exhibits a different system dissipation rate (solid line) compared to that of an uncoupled system (dashed line). (e) Mode 1 dynamics can be qualitatively described by it entering (solid lines) or bypassing (dashed lines) an effective PTS, locking onto the force from mode 2 (see Appendix B). (f) As mode 2 loses energy, the PTS becomes unstable and modes unlock. The simple and intuitive PTS description neglects the nonadiabatic dynamics of mode 2 (see Supplemental Material Note 3 [26]). Compared to the experimental conditions, the schematic of the PTS separatrix uses a much smaller detuning term (see Supplemental Material Note 1 [26]) for clarity of presentation. The experimental phase diagram is presented in Appendix D.

Figure 1(a) depicts two isolated low-loss vibrational eigenmodes of a nonlinear micromechanical resonator used in this study, having frequencies $\omega_{2}/\omega_{1}\approx 3$, and coupled by a cubic nonlinear term [24,25]. The lower-frequency mode 1 and the higher-frequency mode 2 have independent dissipation rates to the thermal bath, ${\text{Γ}}_{1}$ and ${\text{Γ}}_{2}$, and can exchange energy at a rate ${\text{Γ}}_{\text{ex}}\gg {\text{Γ}}_{1},{\text{Γ}}_{2}$. Mode 1 exhibits a pronounced stiffening Duffing nonlinearity, and mode 2 is nearly linear [seeFig.1(b)]. By independent actuation and detection at two separate frequencies, we can prepare the system at prespecified initial amplitudes [solid dots in Fig. 1(b)] and observe each mode’s evolution toward thermal equilibrium shown as the black and red arrows (see Appendix A).

We find that, starting from the same mode energies and depending on the initial relative phase, the system follows one of two radically distinct relaxation pathways leading to different system energy and oscillation frequency $\omega_{1}(t)$ during ringdown [Figs. 1(c) and 1(d)]. One pathway is characterized by a phase-locked state at the 1∶3 internal resonance with a lifetime much longer than the longest thermalization time in the system [solid blue line in Fig. 1(c)]. The distinctive feature of this system state is the lack of decay in the amplitude and frequency of mode 1 supported by a constant rate of energy transfer from mode 2. The intermodal energy transfer and the distinct dissipation rates of the modes lead to different system energy-loss rates along the two pathways shown in Fig. 1(d).

From the perspective of mode 1, the nonlinear coupling with mode 2 manifests as an applied force modulated at $\omega_{2}\approx 3\omega_{1}$; thus, the equation of motion for mode 1 can be generally written as (Appendix H)

(1) $${\ddot{q}}_{1}+{\text{Γ}}_{1}{\dot{q}}_{1}+\omega_{1}^{2}\left(q_{1}^{2}\right)q_{1}=q_{1}^{2}F_{2}(t)\cos\left[\phi_{2}(t)\right],$$

where $q_{1}$ is the modal displacement, and $\omega_{1}\left(q_{1}^{2}\right)$ accounts for the mode’s nonlinearity. Our mode 1 is well described by a Duffing oscillator with a nonlinear coefficient $\alpha_{1}:\omega_{1}^{2}\left(q_{1}\right)=\omega_{1,\text{linear}}^{2}+\alpha_{1}q_{1}^{2}$. To obtain the 1∶3 resonant coupling, the interaction with mode 2 is described by a nonlinear force term $\propto q_{1}^{2}q_{2}$, which in Eq. (1) is represented by the effective drive $F_{2}(t)\cos\left[\phi_{2}(t)\right]\propto q_{2}(t)$. If the nonlinear coupling can be treated as a perturbation, under the conditions discussed in Appendix H, mode 1’s effect on mode 2 phase $\phi_{2}(t)$ can be neglected, and mode 2 performs as a ringdown Duffing oscillator with gradually varying amplitude influenced by slow energy exchange with mode 1. In this limit, the amplitude $F_{2}(t)$ and the frequency $\omega_{2}(t)={\dot{\phi }}_{2}(t)$ vary slowly compared to the mode 1 dynamics, and the fully coupled equations of motion can be reduced to Eq. (1) for mode 1 subject to a period-three drive from mode 2. As we discuss below, the amplitude evolution of both modes on the long timescales of their damping rates can be quantitatively described by accounting for the energy exchange between them.

A form of Eq. (1) for a Duffing oscillator with constant $F_{2}$ and $\omega_{2}$ has been a subject of recent theoretical interest [23–25] due to the existence of a stable phase-locked PTS in the system occurring when the drive-force magnitude exceeds a certain nonzero threshold: $F_{2}>F_{2,\text{threshold}}$[24]. As we experimentally demonstrate below, the dynamics of our fully coupled two-mode system can be well described by the PTS model for the lower-frequency mode 1 under the influence of mode 2 of gradually changing amplitude and near-constant frequency. Furthermore, this model may form a suitable basis for describing the dynamics of a variety of low-loss coupled nonlinear systems (see Supplemental Material Notes 2 and 3 [26]).

Such PTSs for mode 1 are schematically illustrated as solid red dots on the phase diagram shown in Figs. 1(e) and 1(f) (see Appendix B). The sizes of the PTS basins of attraction (color-filled areas) are determined by the magnitude of the modulating force, i.e., the amplitude of mode 2 [27]. For a substantial mode 2 amplitude, Fig. 1(e), the PTS basins of attractions occupy much of the high-amplitude portion of the phase diagram, and the excited system is more likely to fall into the phase-locked state (solid black line) rather than bypassing it (dashed black line). Once mode 1 is locked in a PTS, it remains there until the amplitude of decaying mode 2 becomes so small that the PTS disappears [Fig. 1(f)]. (See Supplemental Material Video 1 and Note 1 [26] for mode 1 evolution following adiabatically decreasing mode 2 amplitude.)

## III. MEASUREMENT OF RELAXATION DYNAMICS NEAR INTERNAL RESONANCE

### A. Phase-locked state at internal resonance

Our experimental system is a vibrating clamped-clamped micromechanical beam that exhibits a fundamental in-plane flexural mode (mode 1) that can couple to a higher-frequency out-of-plane torsional one (mode 2) [11,21]. After preparing the system in an initial excited state, we measure the motion of both modes during subsequent free relaxation (see Appendix C), conducting two series of experiments using different initial states.

**FIG. 2.**
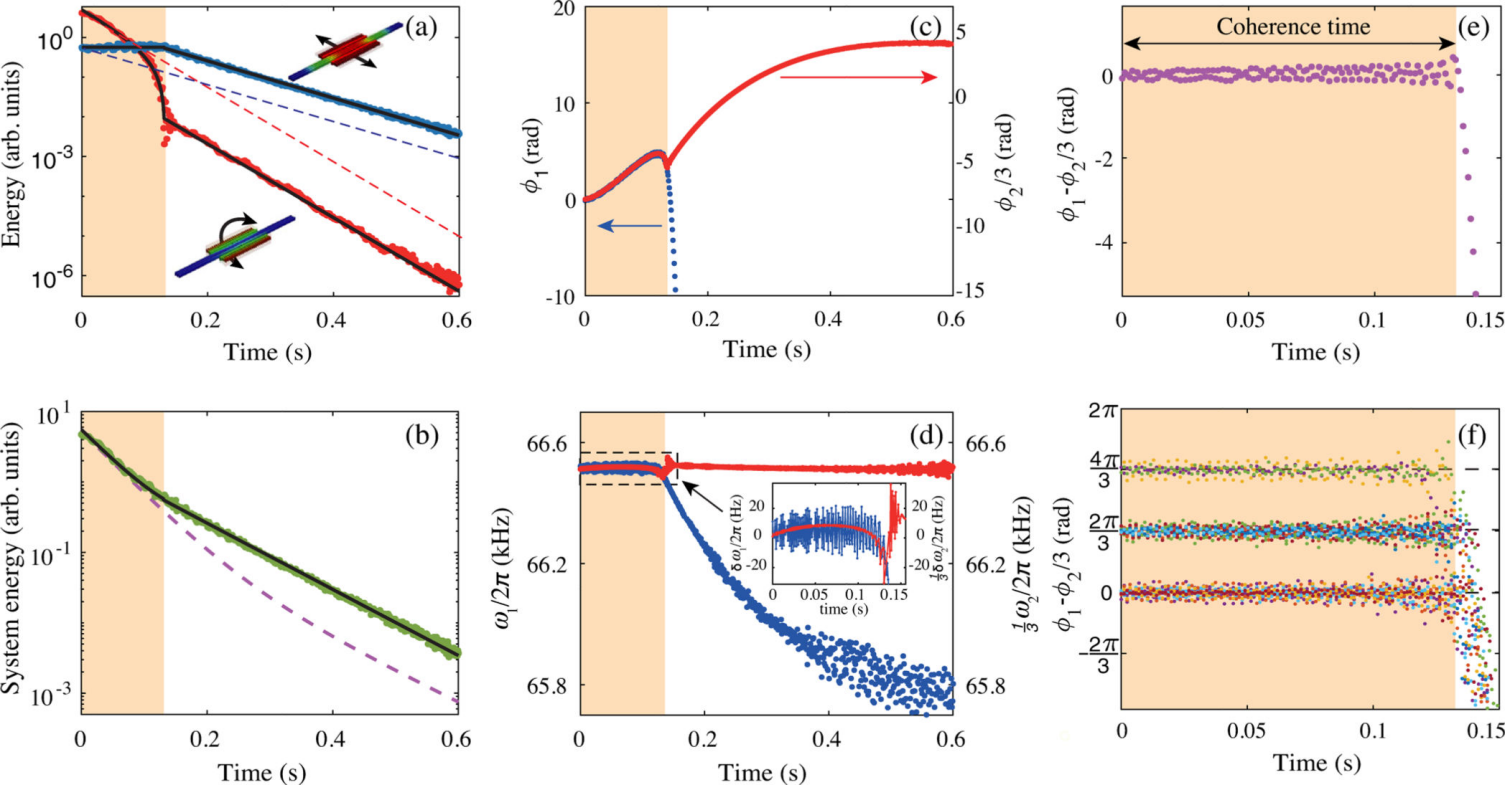
Measured relaxation dynamics of mode 1 and mode 2 from a phase-locked state. (a) Energies $E_{1}$ (blue) and $E_{2}$ (red) for modes 1 and 2, respectively, are plotted on a common scale calibrated from the measured amplitudes. The black lines are fits based on energy transfer between modes. The dashed lines are hypothetical uncoupled energy decays calculated with separately measured individual mode relaxation rates. The yellow highlighted area represents the phase-locked time period. (b) Measured system energy $E_{1}+E_{2}$ (green dots), and the model fit (black line), with the hypothetical uncoupled (purple dashed line) decay for comparison. (c) Measured phase of mode 1 (blue dots) and mode 2 (red dots) with reference frequency of $\omega_{2\;\text{linear}}/3$ and $\omega_{2,\text{linear}}$, respectively. We define $\phi_{2}(t=0)=0$ as a start phase. (d) Measured frequency. Inset shows the enlargement of the dashed area, where $\delta \omega_{1}=\omega_{1}-\omega_{2\;\text{linear}}/3$ and $\delta \omega_{2}/3=\left(\omega_{2}-\omega_{2,\text{linear}}\right)/3$. (e) Measured relative phase $\phi_{1}-\phi_{2}/3$ is constant in the phase-locked state. (f) Repeat measurement of relative phase $\phi_{1}-\phi_{2}/3$ for 15 times. They show signature feature of the 2π/3 relative phase difference for the period-tripling states.

In the first series, we prepare the system initially in the phase-locked state by solely driving mode 1 at the internal resonance (see Appendix A). During the subsequent free ringdown, as shown in Fig. 2(a), there is a finite period of time, the “coherence time” $t_{c}$ (colored area in Fig. 2), where the energy of mode 1 remains approximately constant (variation <16%) while mode 2 loses energy much faster than its dissipation rate into the thermal bath. Beyond $t_{c}$, the modes unlock from each other and follow their individual exponential decays toward equilibrium [11,12].

The observed evolution of the amplitudes is well described by a simple model, which assumes that each mode’s coupling to the thermal bath is independent and linear, and the full system energy is the sum of the energies of individual modes (i.e., time-averaged interaction energy is negligible). This condition is a good approximation for our system, though it does not generally apply to internal resonance systems. Each mode energy is assumed proportional to the square of the amplitude of the fundamental harmonic oscillation, $E_{1(2)}\propto A_{1(2)}^{2}$.

During $t_{c}$, the conservation of energy equates the extra power lost by mode 2 to the power received by mode 1 after accounting for their intrinsic losses. The exchange power W is constant because it exactly offsets the dissipation of mode 1, maintaining a constant amplitude on its Duffing curve at the locked frequency of $\omega_{2}$=3 during $t_{c}$. We therefore can simultaneously fit both mode amplitudes, calibrate them, and plot them on a common energy scale using only one fitting parameter W (see Appendix G).

In Fig. 2(b), we plot the experimentally measured system energy as a function of time. For comparison, the dashed line corresponds to the expected energy-relaxation trajectory for the system without coupling between the modes. The slower relaxation of the coupled-mode system can be explained by noting that in our system mode 2 has a higher loss rate $\left({\text{Γ}}_{2}/2\pi \approx 3.3\;\text{Hz}\right)$ than mode 1$\left({\text{Γ}}_{1}/2\pi \approx 1.5\;\text{Hz}\right)$, and thus, the energy flow from a higher-loss-rate mode to a lower one decreases the overall system dissipation.

The temporal evolution of each mode’s phase and frequency are shown in Figs. 2(c)–2(e). A key observation is that the modes remain phase locked for the duration of $t_{c}$ [Fig. 2(c)], and, as a consequence, the relative phase $\phi_{1}-\phi_{2}/3$ remains constant at around 0 rad for this experiment [Fig. 2(e)], corresponding to one of the three PTS states [Fig. 2(f)]. After coherence time, the modes unlock, and each mode frequency evolves toward the corresponding small-amplitude eigenfrequency, $\omega_{1,\text{linear}}/2\pi \approx 64630.6\;\text{Hz}$ and $\omega_{2,\text{linear}}/2\pi \approx 199881.8\;\text{Hz}$.

**FIG. 3.**
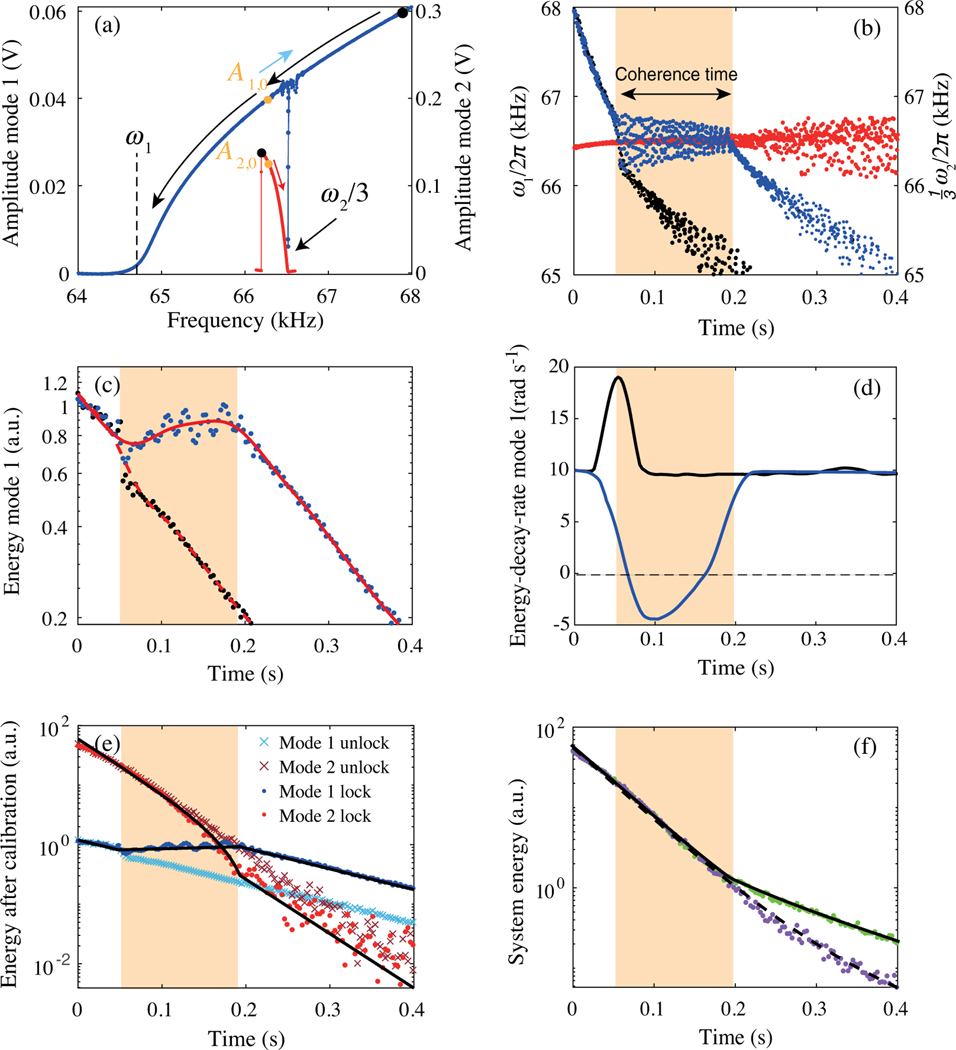
Measured relaxation dynamics of mode 1 and mode 2 from an initially uncoupled high-energy state. (a) The modes are prepared at the initial amplitudes marked by black dots by independently driving them along the shown trajectories. With the drives off, following an initial free decay, the modes reach internal resonance at the amplitudes labeled by green dots, and either enter or bypass the phase-locked state, depending on their relative phase. The arrows show the mode 2 amplitude-frequency trajectory (red arrow) and the mode 1 trajectory for the unlocked (black arrow) and the locked (blue arrow) states. Both modes’ amplitude-frequency trajectories are confined to their respective individual nonlinear eigenfrequency-amplitude relationships. The narrow dip on the amplitude-frequency curve of the driven mode 1 corresponds to crossing the 1∶3 internal resonance at $\omega_{2}$/3, while the drive to mode 2 is off. (b) Oscillation frequency of locked mode 1 (blue dots) and mode 2 (red dots), with comparison to that of the unlocked mode 1 (black dots). (c) Energy of mode 1. Blue and black dots correspond to the locked and unlocked cases. The amplitude oscillations corresponding to the frequency oscillations apparent in (b) are small due to the large Duffing term (see Appendix D) and are not visible in the energy data downsampled to reduce noise. The dashed (solid) line is a 0.03-s moving average of the unlocked (locked) data. (d) Energy-decay rate of mode 1 extracted from the averaged locked (blue) and unlocked (black) data in (c). The increasing energy of mode 1 is supplied by mode 2. (e) Two distinct energy-relaxation trajectories with different final energies from the same initial energies for mode 1 and mode 2. (f) System energy pathways. Black lines are obtained from the calibration based on energy transfer between modes (see Appendix G).

Figure 2(d) shows the measured frequencies of the modes during and after coherence time. As previously mentioned, when the modes are locked, the frequencies of both mode 1 and mode 2 remain essentially constant (relative change < ±0.05%). In the internal resonance, mode 1 exhibits small oscillations (approximately 10 Hz root-mean-square) around the smoothly varying $\frac{1}{3}\omega_{2}/2\pi \approx 66.5\;\text{kHz}$. The amplitude oscillations corresponding to the frequency oscillations apparent in Fig. 2(d), and similarly Fig. 3(b), are small due to the large Duffing term (Appendix D) and cannot be resolved. Mode 2 shows gradual changes smaller than approximately 10 Hz (approximately 0.015%), and a larger frequency “pull” and “rebound” of ±30 Hz (approximately ±0.05%) at the unlocking point. Away from the unlocking point, these minor mode 2 deviations could be attributed to its small softening nonlinearity and off-resonant nonlinear interactions.

Since the frequency of mode 2 remains essentially constant throughout the internal resonance, we posit that here mode 2 can be considered an adiabatically changing external nonlinear driving force, locking mode 1 into one of three PTSs (see Appendix H). As the system loses energy, the amplitude of mode 2 decreases, shrinking the PTS basin of attraction, and when its amplitude drops below a fixed threshold $F_{2\;\text{,threshold}}$, the PTS disappears [Figs. 1(e) and 1(f)].

We repeat the ringdown measurement multiple times and find that the mode 1 randomly locks to one of the three states, resulting in three different relative phase. Figure 2(f) shows the relative phase of 15 groups of ringdown data with nearly the same coherent time. Their relative phase shows discrete distribution as shown in the PTS diagram shown in Fig. 1(e).

### B. Nonmonotonic energy dependence of dissipation rate and phase-dependent relaxation pathways

One important advance in this work is that we have full experimental access with sufficient sensitivity and band-width to measure the dynamics of higher-frequency mode 2, and, therefore, the relative phase, as well as the system energy, which could not be done in the previous studies [11,12,21]. Another important advance is the ability to independently control the initial conditions of both mode 1 and mode 2. As PTS is largely determined by the dynamics of mode 2$\left(F_{2}\propto q_{2}\right)$, including the relative phase as well as the amplitude, the independent initial condition control is important for understanding how the parameters of mode 2 influence the relaxation of mode 1, and thus the system’s pathway toward equilibrium. For this purpose, in the second series of experiments, we prepare the system in the unlocked initial state with starting energies above the internal resonance [black dots on the two Duffing curves in Fig. 3(a)]. After this initial state is prepared by sequentially sweeping the two drive signals (see Appendix A), they are turned off simultaneously, and the motion of both modes is recorded.

Since mode 1 and mode 2 have Duffing characteristics with opposite sign, $\omega_{1}$ and $\omega_{2}$ reach $\omega_{1}:\omega_{2}=1:3$ at particular amplitudes $A_{1,0}$ and $A_{2,0}$ during the ringdown, as indicated by green dots in Fig. 3(a). Before that, the modes relax independently with their frequencies changing according to their amplitude-eigenfrequency curves [Fig. 3(b)]. Surprisingly, when the frequencies become commensurate, phase locking does not always occur. We repeat the experiment up to 100 times with the same initial amplitudes but arbitrary relative phases. Once the frequencies reach the internal resonance, mode 1 frequency either locks to mode 2 (blue dots) or bypasses it (black dots). These results indicate that the relative phase among modes is key for entering the phase-locked state, as shown schematically in Fig. 1(e) and with experimental data in Appendix D, Fig. 8(e). When the modes lock, mode 1 frequency is observed to oscillate with gradually decreasing amplitude around the mode 2 frequency, as mode 1 settles toward the PTS.

Figure 3(c) shows the mode 1 energy for the phase-locked and unlocked cases. When the modes lock, the energy can flow between them, while the time-averaged interaction energy remains low compared to the individual mode energies—no abrupt frequency shifts are observed at the locking and unlocking times, and the modes continue to follow their individual amplitude-frequency relationships. In the locked state, the net system energy continues to decrease through dissipation, while the frequencies of both modes evolve together in the direction that lowers the net energy, following their frequency-amplitude relationships. Here, $\omega_{1}=\omega_{2}/3$ increase together and mode 2 amplitude drops, while mode 1 amplitude increases with time [Fig. 3(a) blue arrow].

Separate from the oscillations observed in mode 1 frequency as it settles toward the PTS, small slow variations in the energy of mode 1 are observed, possibly arising from the nonadiabatic evolution of the pseudopotential. The resulting dynamic energy exchange between the modes is not described by the simple PTS model, requiring further investigation. For the case where locking is bypassed (black dots), a sharp loss rate increase is visible at the bypassing of internal resonance, consistent with the PTS model (see Supplemental Material Note 1 [26]). Qualitatively, for a certain range of the relative phase, energy transfers away from mode 1 to mode 2, therefore briefly accelerating the mode 1 decay.

The measured time-averaged energy-decay rate of mode 1 is shown in Fig. 3(d) for the locked (blue) and unlocked state (black). As it is evident from the data, the coupling among modes qualitatively changes the effective energy-decay rate of mode 1: It exhibits a striking nonmonotonic energy evolution with a negative dissipation rate. When the modes do not lock, the energy loss of mode 1 remains positive during the entire relaxation time, with a transient peak observed when the frequencies cross the internal resonance. This transient rapid energy loss observed during bypassing is distinct from the sustained faster energy loss observed at higher amplitudes in Ref. [12]. It indicates that the energy gain and loss shown here and Ref. [12], respectively, are not due to their possibly different initial conditions.

The energies of both modes are displayed in Fig. 3(e) and their sum in Fig. 3(f). The data clearly demonstrate that there are two distinct energy-relaxation pathways for the system and that they are determined solely by the initial relative phase between the two modes (Appendix D). Furthermore, because of the ability of the system to dynamically shift the energy between multiple modes with different modal dissipation rates, the system energy dissipation becomes a nonlinear function of the net system energy for as long as the modes are locked.

### C. Tunable coherence time and phase-lock probability

**FIG. 4.**
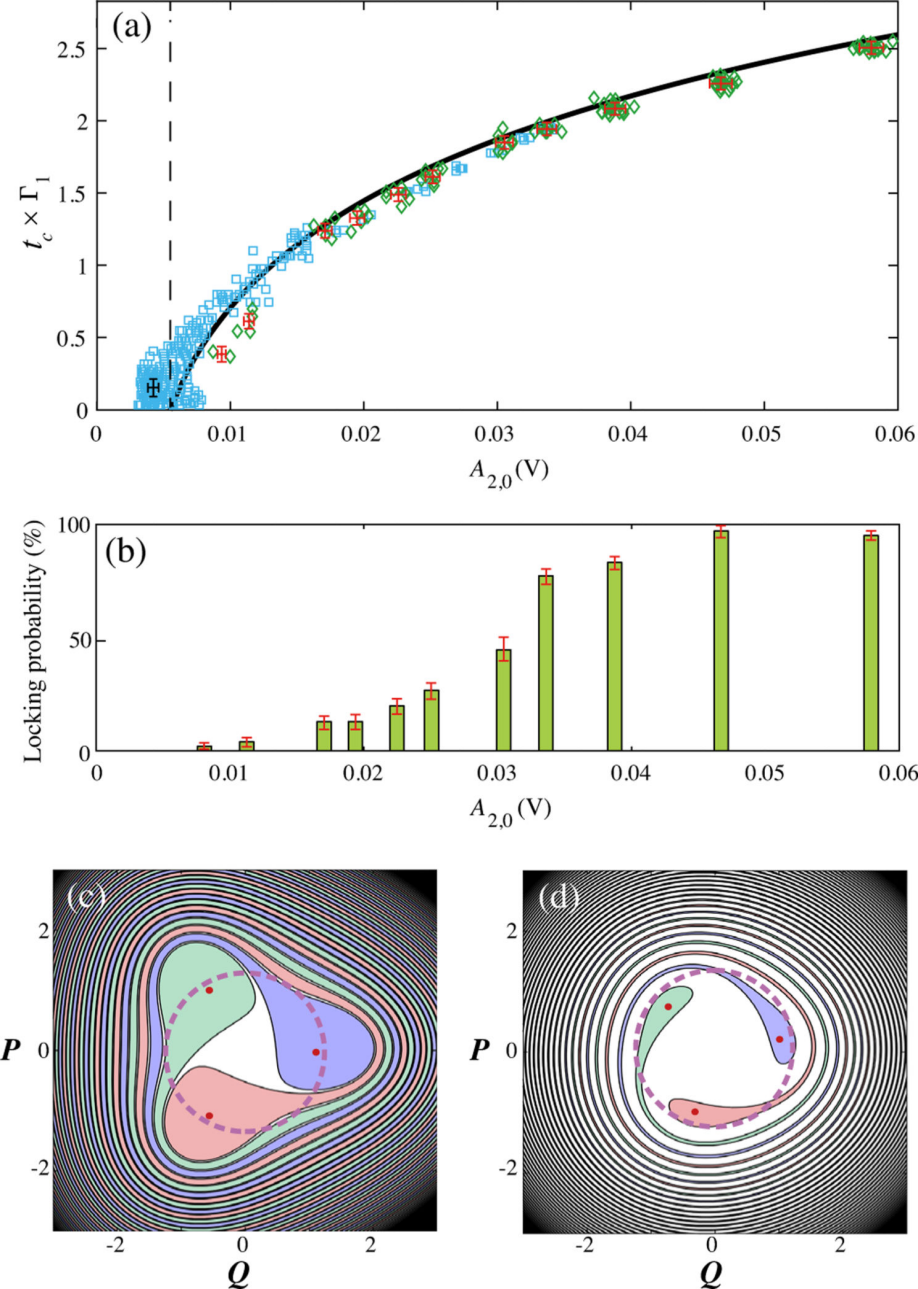
Coherence time and the probability of locking. The coherence time and the locking probability increase as a function of the mode 2 amplitude $A_{2,0}$ at the initial time of locking and are independent of all other parameters. (a) Measured $t_{c}$ in the units of mode 1 decay time $1/{\text{Γ}}_{1}$. Blue squares are experiments preparing mode 1 and mode 2 at the phase-locked state initially, while green diamonds are measured when the modes are initially unlocked. The black dashed line $A_{2\;\text{,threshold}}$ ≈0.006 V is obtained from the averaged $A_{2,0}$ at $t_{c}\approx 0\;\text{s}$. The black calculated coherence time is obtained via the calculated relaxation time of mode 2 from $A_{2,0}$ to $A_{2\;\text{,threshold}}$ without using any adjustable parameters. The uncertainties for the blue squares are the same for each data point shown by the single black cross, while the uncertainties for points with in each group of the green diamonds are labeled on the corresponding data groups. Uncertainty definitions and analysis are given in Appendix F. (b) The probability for locking increases as a function of $A_{2,0}$. The results are from 100 measurements with random initial relative phase $\phi_{1}-\phi_{2}/3$ for each $A_{2,0}$. The uncertainties are 1 standard deviation of the corresponding binomial distribution. (c) PTS basins of attraction for strong driving strength. (d) PTS basins of attraction for weak driving strength. The purple dashed rings represent the initial condition of mode1 with a fixed amplitude $A_{1,0}$ slightly above the PTS (red dots) and a random relative phase. Starting points in the shaded (white) areas fall into the locked PTS states (unlocked trivial state). Larger mode 2 amplitude increases the combined are as of the PTS basins of attraction. Compared to the experimental conditions, the schematic of the PTS separatrix uses a much smaller detuning term (see Supplemental Material Note 1 [26]) for clarity of presentation. The experimental phase diagramis presented in Appendix D.

**FIG. 8.**
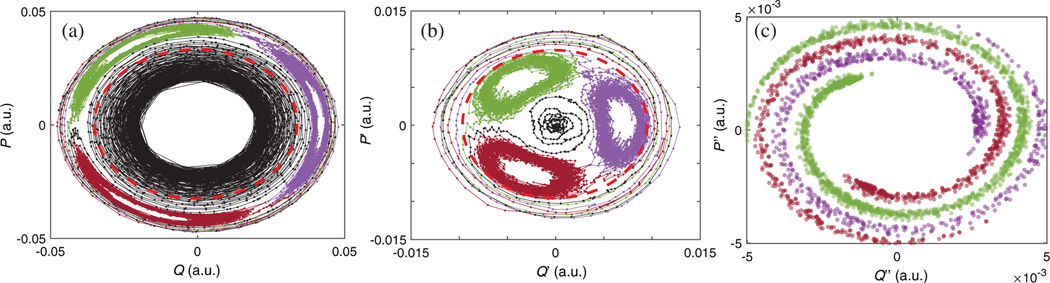
The dynamics of mode 1 in the PTS pseudopotential with a reference phase of $\phi_{2}(t)/3$. (a) Four groups of locking and bypassing data taken at the same experimental conditions as shown in Fig. 3. (b) Same as (a) but rescaling the radial scale by setting origin at R=0.033 [red dashed circle in (a)] instead of 0. Three groups of data lock and precess around three PTS stationary points, while one group bypasses and rings down to the ground state. (c) 44 groups of experimental data that result in locking out of 100 repeated experiments with uniformly distributed random initial phases. They are colored based on which basin they end up with. The other 56 groups of data (not shown) are all bypassing the lock. The bypassing data concentrate within the white space between the colored spirals. It is comparable to Fig. 4(d). In (c), we focus on the data just before entering the basin regions by further enlarging and setting the origin at R=0.040, i.e., $Q^{\prime \prime }+iP^{\prime \prime }=(R-0.040)e^{i\varphi }$ and truncating the data traces to include only R>0.042 [red dashed line in (b)].

With the added flexibility of controlling the initial conditions of the two modes independently, we observe different coherence times. We discover that $t_{c}$ can be controlled by changing the amplitude of mode 2 at locking $A_{2,0}$ and is nearly independent of the other state variables (see Appendix G). Figure 4(a) shows the measured $t_{c}$ normalized by the longest thermalization time constant $1/{\text{Γ}}_{1}$ in the system for different $A_{2,0}$. The blue squares correspond to the coherence times measured when mode 1 is prepared initially at the internal resonance (data shown in Fig. 2), and the green diamonds show the measured coherence times when modes 1 and 2 decay from an initially unlocked state (data shown in Fig. 3). Among all our experiments, which differ by their initial states, $t_{c}$ is fully determined by $A_{2,0}$, independent of any other variables describing the state of our system. Indeed, the $t_{c}$ is given by the time needed for $A_{2}$ to decay from $A_{2,0}$ to a threshold amplitude $A_{2\;\text{,threshold}}$, a minimum amplitude required to maintain PTS, which is a property of the system independent of its state. As $A_{2,0}$ is controllable, $A_{2\;\text{,threshold}}$ is directly measured [black dashed line in Fig. 4(a)], and the energy dissipation of mode 2 is fully captured based on energy transfer between modes [fits in Figs. 2 and 3 using Eq. (G2)]. $t_{c}$ can be obtained via calculating the ringdown time of mode 2 from $A_{2,0}$ to $A_{2\;\text{,threshold}}$, Eq. (G3) (see Appendix G). The calculated coherence time is shown as the black line in Fig. 4(a) without using any adjustable parameters.

While the tunable $t_{c}$ and nonmonotonic energy-dependent dissipation rates offer a novel strategy for dissipation engineering, the probabilistic nature of reaching a phase-locked state during thermalization from a random relative phase must be taken into consideration. When the modes are relaxing from an unlocked state, the initial relative phase of the modes can be set independently from other parameters. Figure 4(b) shows the statistical results for 100 energy-decay measurements under random initial relative phase conditions for each $A_{2,0}$. The data indicate that a minimum $A_{2,0}=A_{2\;\text{,threshold}}$ is required to reach the phase-locking condition and that the likelihood of locking increases with $A_{2,0}$ approaching unity for large amplitudes. These observations are in agreement with the PTS-based [24,26] model, where the basins of attraction for the stable states become larger with increasing $A_{2,0}\propto F_{2}$. Figures 4(c) and 4(d) are schematics of the PTS basins of attraction for large and small $A_{2,0}$, respectively. The purple dashed circle depicts the mode 1 states with a given amplitude and random initial relative phases. For larger $A_{2,0}$, the majority of the points on the ring lie in the color-filled PTS basins of attraction, and thus, the chance for mode 1 to fall into a PTS is significantly larger.

## IV. CONCLUSION AND OUTLOOK

A wide class of low-loss systems can be described by a set of independent nonlinear eigenmodes exchanging energy when two or more of their frequencies match, forming integer multiple relationships. In contrast to trivial brief energy exchanges occurring when the dynamically evolving eigenfrequencies intermittently match, here, using a simple, fully observable, two-mode system, we show how eigenmodes can phase lock at internal resonances and remain locked for time periods up to 6 times longer than the system relaxation times (i.e., $1/{\text{Γ}}_{2}$). This allows the modes to rapidly exchange large amounts of energy, producing nonmonotonic modal energy evolution, making the locked system energy dissipation nonlinear, and resulting in completely different energy states compared to when the phase locking has been bypassed. While locked, the energy exchange rate depends on the individual mode’s eigenfrequency-energy relations and dissipation rates but does not significantly depend on the exact value of the coupling between them.

We show that entering or bypassing the phase-locked state depends sensitively on the initial relative phase, and thus the system’s final state is a high-gain nonlinear amplifier and discriminator of the relative phase and relative frequency. This phase dependence distinguishes the observed behavior from nonlinear damping recently observed in driven two-mode systems [28].

The experimentally measured relaxation dynamics of the two-mode system, including phase locking, is completely described by extending the well-understood model of a single-mode system subject to a commensurate nonlinear drive, e.g., the period tripling state of a Duffing oscillator. The PTS model is accurate when the higher-frequency-mode dynamics are slow compared to the mode 1 precession frequency in the PTS pseudopotential, i.e., when mode 2 undergoes gradual energy change and the corresponding slow frequency shift according to its Duffing nonlinearity. This condition is clearly valid for our experiment and would be valid for many other systems with low natural decay rates. Additionally, for a system with small time-averaged interaction energy relative to the modes’ energies, the long-timescale energy evolution can be modeled by a single-parameter energy-transfer equation [Eq. (G1)] providing an intuitive picture simplifying the general fully-coupled-equation-of-motion description (Appendix H). While phase locking is predicted within the PTS model, more general theoretical approaches [13] are needed to study the complete parameter range where the discovered phase-locking phenomenon may be observed outside the validity range of the PTS model.

Numerical simulation of our PTS model can qualitatively reproduce the contradictory experiment results of the rapid energy loss [12], gain [11,21], and nonmonotonic energy (this work) using different effective Duffing coefficients of mode 2 (Supplemental Material Note 2 [26]). However, we cannot confirm that our model is applicable to the specific experimental systems other than the one studied here. More complex phenomena could also lie at the root of such contradictory results. Within this model, the direction of the energy exchange depends on the specifics of each mode’s eigenfrequency-energy relationship near the internal resonance and can be the same as or opposite to the one observed in the present experiments. For example, mode 2 with a linear (zero) eigenfrequency-energy relationship can lock mode 1 at a constant frequency and amplitude as shown in Fig. 2, while mode 2 with a hardening eigenfrequency-energy relationship can drag the locked mode 1 to decay even faster than its natural relaxation rate, opposite to the energy gain effect shown in this work. Beyond the coupled Duffing oscillators, this model can be extended to describe two modes with arbitrary nonlinearities near an internal resonance (Supplemental Material Note 3 [26]).

Recently, the authors learned that the energy transfer between coupled modes at 1∶3 internal resonance was studied by Yan *et al.* [29] Complementing the present study of the transient relaxation, Yan *et al.* independently demonstrate that the same period-tripling approach quantitatively describes the steady-state dynamics of such systems subject to a continuous external drive of the higher-frequency mode. Demonstrating the rich dynamics possible in such systems, when the nonlinear second mode is driven sufficiently strongly, it responds with one of two distinct amplitudes. Taken together with the period tripling for the lower mode, this combination leads to a total of six distinct discrete time translation symmetry breaking states. Parametric 2f drive for mode 2 also results in six coexisting states.

A single nonlinear mode can be driven parametrically at arbitrary frequency multiples. Similarly, pairwise mode locking can occur at resonances of different integer fractions, other than the 1∶3 studies here. Moreover, the proposed description framework may be fruitfully applied not only to driven systems [29] but also to systems with more than pairwise resonances, i.e., when more than two modes (and/or drives) participate in the resonant energy exchange (Supplemental Material Note 3 [26]). While, generally, much richer, such systems may exhibit extended locked states strongly influencing their dynamics under some conditions. Our work opens up a new window for exploration and harnessing of such a general class of nonlinear systems, both in and out of thermal equilibrium. Applying them to many-body systems with large numbers of coupled elements could lead to additional interesting applications, such as tailoring the energy flow direction and efficiency [9] and the broadband sensing with large assemblies of coupled oscillators [30]. These results are not limited to nonlinear mechanics and shed light on coupled nonlinear resonator systems across physical domains, including, for example, nonlinearly coupled optical modes in chip-scale photonic resonators.

## Supplementary Material

Supp1

## Figures and Tables

**FIG. 5.**
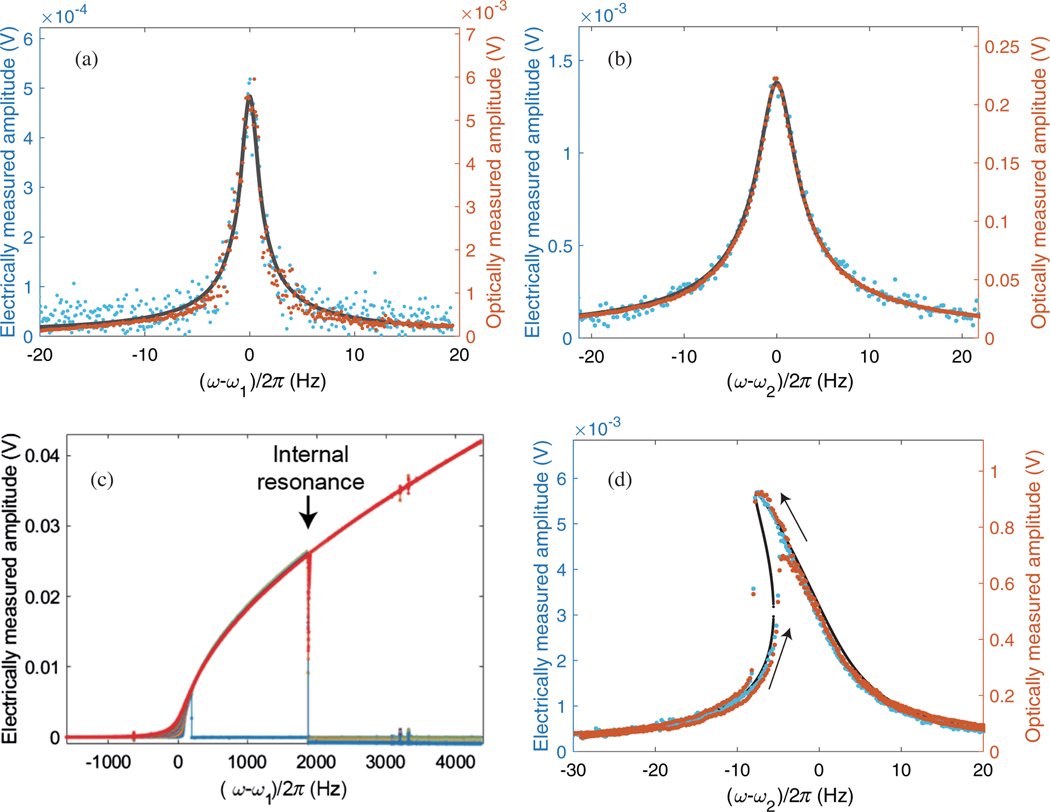
Two mechanical modes with nearly 1∶3 commensurate frequency relationship. (a) Linear, small-amplitude resonance of mode 1. The blue and orange dots present the measured data from the electrical and optical detection, respectively. The black line is from the Lorentzian fit. (b) Linear, small-amplitude resonance of mode 2. (c) Nonlinear response of mode 1 for the ac component of the diving voltage ranging from 0.01 to 0.81 V with a 0.05 V increment. (d) Nonlinear resonance of mode 2 with a hysteresis. The black line is a Duffing model fit. The arrows label the sweeping directions.

**FIG. 6.**
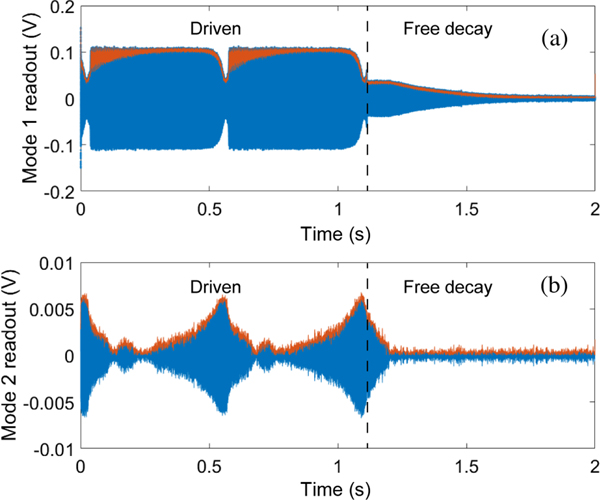
Preparation of the initial state at the internal resonance. Electrically measured displacement of (a) mode 1 and (b) mode 2. A limit cycle under a steady drive is followed by the free decay of the coupled system after the drive is turned off (black dashed line) near mode 2’s maximum amplitude. The orange line labels the vibration amplitude. The electrical capacitive measurement gain for torsional mode 2 is much smaller than for translational mode 1.

**FIG. 7.**
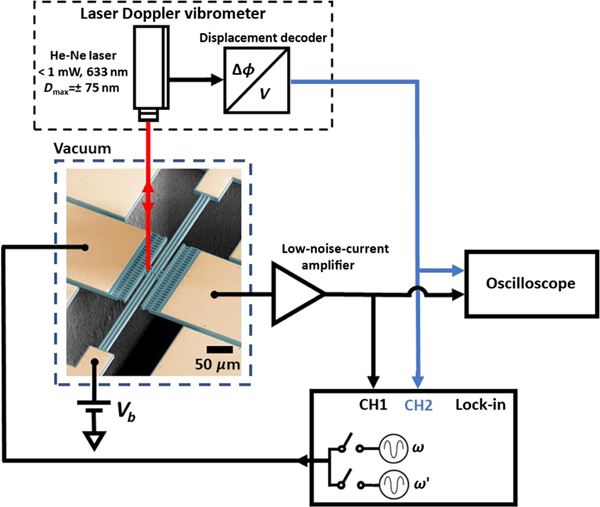
Measurement setup: The schematic shows the clamped-clamped beam resonator and the wiring of the capacitive (black) and optical (blue) detection. In addition to the electrical measurement through the lateral comb electrode, we measure the out-of-plane mode using a laser Doppler vibrometer.

**FIG. 9.**
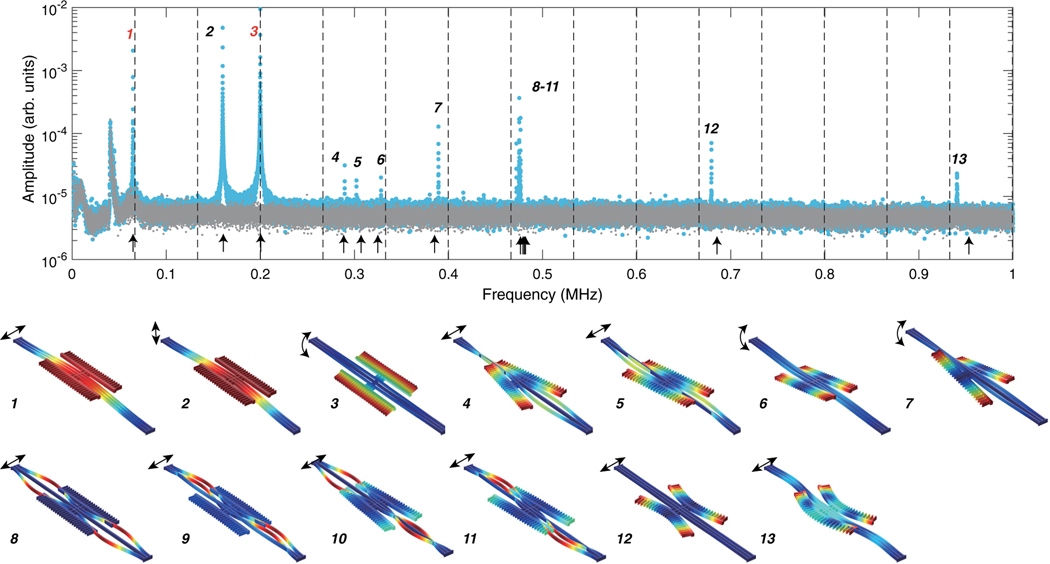
Measured mechanical spectrum (blue dots) and simulated mode shape are shown as upper and lower panels. The simulated (up-pointing black arrows) and measured eigenfrequencies match each other, with disagreement <5% due to fabrication imperfections. Dashed lines are integer multiples of the internal resonance frequency. They indicate there is no other matching frequency at the 1∶3 internal resonance. The gray dots are the measurement noise floor. The arrows in the modal shape panel depict in-plane flexural modes, out-of-plane flexural modes, and torsional modes. Modes 8 to 11 are four degenerate beam modes.

**FIG. 10.**
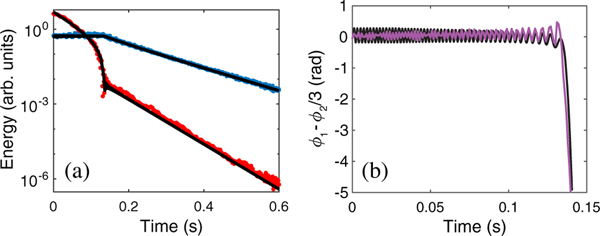
Fit by complete model. The fitted results are shown as black lines for amplitude (a) and phase (b). The red and blue symbols and the purple line are the experimental data. The complete model, i.e., Eqs. (H5) and (H6), agree with the measured energy data as well as the time-averaged energy-evolution fit in Fig. 2(a) from the simple model. Beyond that, the complete model reproduces the experimentally observed fast phase oscillation as the mode 1 rotates in the PTS pseudopotential with precession frequency Ω, also shown in Video 1 in the Supplemental Material [26].

**FIG. 11.**
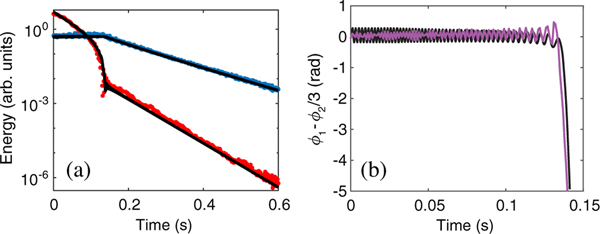
Modeling results with a single coupling parameter as the sole fit parameter, using the previously obtained calibration to a common energy scale. The labels are the same as Fig. 10; the only difference is that we fix $g_{21}=8.8g_{12}$ and fit a single parameter *g*_12_. It generates $g_{12}\approx 3.07\times 10^{5}{\text{s}}^{-2}{\text{V}}^{-2}$ and $g_{21}=8.8g_{12}\approx 2.70\times 10^{6}{\text{s}}^{-2}{\text{V}}^{-2}$.

**FIG. 12.**
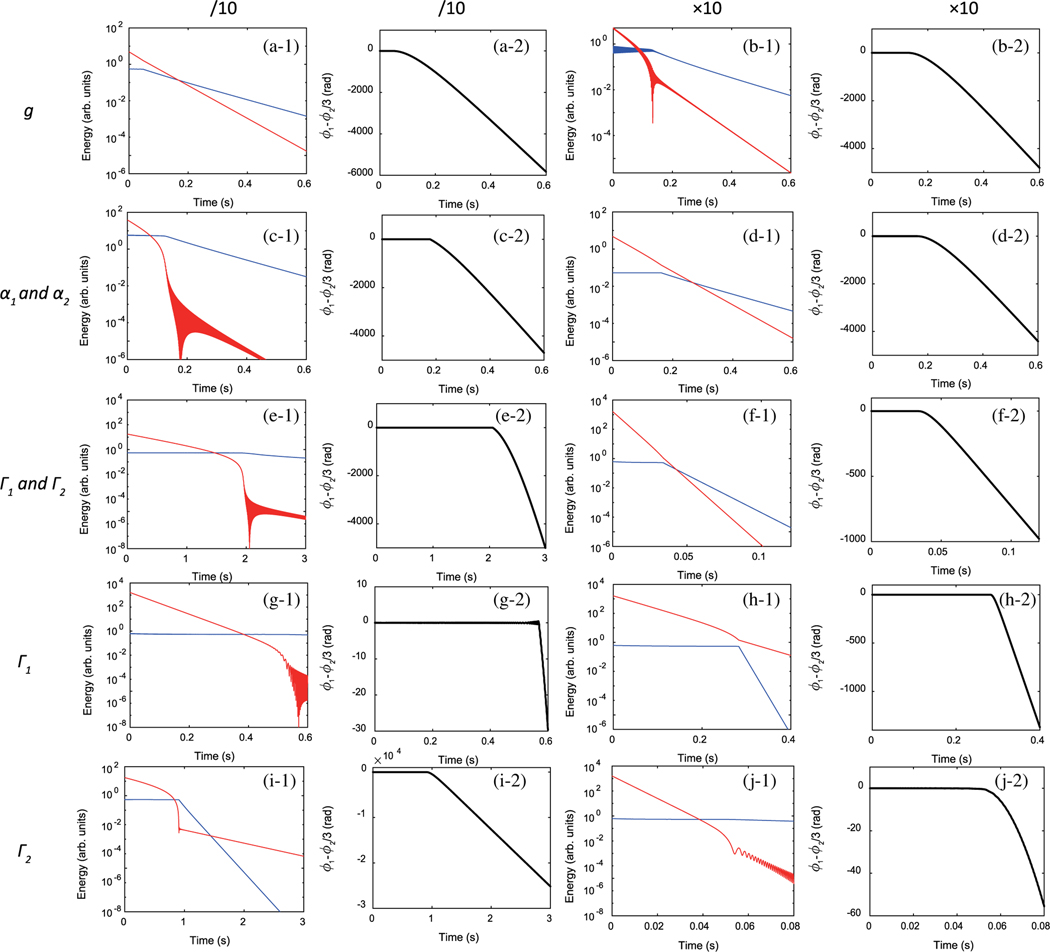
Simulated energy and relative phase of the two modes using the fully coupled two-mode model with parameters scaled as follows relative to the ones used for Fig. 11: g/10 (a), g×10 (b), $\alpha_{1}$/10 and $\alpha_{2}$/10 (c), $\alpha_{1}$ × 10 and $\alpha_{2}$ × 10 (d), ${\text{Γ}}_{1}$/10 and ${\text{Γ}}_{2}$/10 (e), ${\text{Γ}}_{1}$ × 10 and ${\text{Γ}}_{2}$ × 10 (f), ${\text{Γ}}_{1}$/10 (g), ${\text{Γ}}_{1}$× 10 (h), ${\text{Γ}}_{2}$/10 (i), ${\text{Γ}}_{2}$× 10 (j). Within a wide range of the parameters, the persistent locked states are shown. The blue and red lines denote modes 1 and 2’s energy, respectively. The black line denotes their relative phase. When the system is initialized with $E_{2}\gg E_{1}$ and the relative phase near the expected PTS locked state, an extended phase-locked period of time with near-constant mode 1 amplitude is observed for each parameter set.

## APPENDIX A: PREPARATION OF THE SYSTEM’S INITIAL STATE

We use two methods for setting the initial condition of mode 1 and mode 2 in the ringdown experiment. The first method is the same as the one we used previously in the internal resonance research where we drive mode 1 solely to the nonlinear regime and match the drive frequency $\omega_{1}$ to $\omega_{2}$/3 [11,21]. Because of resonantly enhanced energy transfer, energy flows between the two modes periodically, generating a limit cycle [31]. In such a case, the two modes are prepared in the phase-locked state before ringdown; i.e., mode 1 is at the PTS initially. By fine-tuning the turning-off time on the limit cycle, we can control the initial amplitude (energy) of mode 2.

We consider two mechanical modes on the doubly clamped beam. They initially have nearly 1∶3 commensurate eigenfrequencies. Figures 5(a) and 5(b) show the measured linear resonance of mode 1 and mode 2. As we discuss in the previous section, we measure the displacement of the modes both electrically and optically, shown as the blue and orange dots. The Lorentzian fit of the mode shows $\omega_{1,\text{linear}}/2\pi \approx 64\;630.6$ Hz, ${\text{Γ}}_{1}/2\pi \approx 1.5\;\text{Hz}$, and $\omega_{2,\text{linear}}/2\pi \approx 199\;881.8\;\text{Hz}$, ${\text{Γ}}_{2}/2\pi \approx 3.3\;\text{Hz}$. With increasing the driving force, mode 1 turns into a nonlinear regime shown as Fig. 5(c) where a spring-hardening effect is observed (positive Duffing coefficient). When the ac voltage is larger than 0.06 V, the Duffing curve of mode 1 reaches the internal resonance $\left(\omega_{1}:\omega_{2}=1:3\right)$ during the upward frequency sweep. Because of the strong nonlinear intermodal coupling, it cannot pass the internal resonance with mode 2 unless the driving voltage is higher than 0.45 V. For such a voltage, mode 1’s Duffing curve can be swept to higher frequencies shown as the red line, for example. Unlike mode 1, mode 2 shows the spring-softening effect (negative Duffing coefficient) as shown in Fig. 5(d). The fit based on Duffing nonlinearity (black line) describes well the electrically and optically measured mechanical response spectrum of mode 2.

To monitor the energy-decay process of the two modes, we need to prepare the two modes to the preset initial conditions. We use two methods to perform the preparation of the initial state. In the first set of experiments (Fig. 2), we prepare the two modes at internal resonance [black arrow in Fig. 5(c)] by driving only mode 1. The drive frequency is swept up to the internal resonance at $\omega_{2}$/3. The strong nonlinear intermodal coupling transfers energy between mode 1 to mode 2 back and forth, inducing limit cycles. Figures 6(a) and 6(b) present the time-varying amplitudes of mode 1 and mode 2, respectively, at a constant drive amplitude and a fixed frequency near the internal resonance. The amplitudes of mode 1 and mode 2 periodically vary, with short time intervals when energy is exchanged quickly (faster than ringdown times) back and forth between them. We turn the drive to mode 1 off at a specific time, for example, as labeled by the black dashed line. The initial conditions for the two modes at the beginning of the free ringdown are the corresponding values of amplitude at that time. It is noteworthy that although we drive only mode 1, the maximum energy stored in mode 2 can exceed mode 1 energy by up to 10 times at certain points in the cycle shown in Fig. 2(a). In the second set of experiments (Fig. 3), we separately drive mode 1 and mode 2 by two independently controlled drives, by which we can set the initial conditions of the two modes to whatever condition we want. However, later experimental results show that the coherence time strongly depends only on the amplitude of mode 2 at the beginning of locking ($A_{2,0}$).

When the drive to mode 1 at internal resonance is strong, the strong interaction between mode 1 and mode 2 terminates the limit cycle and both modes drop to the ground states. It limits the maximum $A_{2,0}$ that can be obtained in this way, and, therefore, the longer $t_{c}$ shown as blue squares in Fig. 4(a). To obtain even longer coherence time and explore larger parameter space, we use the second method that drives mode 1 and mode 2 via sequential sweeps of two simultaneously applied independent drives. We first sweep the driving frequency of mode 1 to a value much higher than $\omega_{2}$/3 while keeping the drive of mode 2 off. After reaching the high mode 1 frequency, we maintain the mode 1 drive while turning on the drive at mode 2 frequency and sweep it down until mode 2 reaches the desired initial amplitude. As mode 2 has a lower frequency at high amplitude due to its spring-softening effect, the frequency mismatch between the modes is large and interaction is weaker from the internal resonance. We then tune the driving frequency of mode 1 down to the desired value. Two modes maintain their initial amplitudes until we start the free decay by simultaneously turning off the drives [black dots in Fig. 3(a)]. The initially uncoupled modes allow us to study the locking probability and directly observe the two different energy-relaxation pathways of the system.

## APPENDIX B: PHASE DIAGRAM OF PERIOD-TRIPLING STATES

In this specific experiment, the mode nonlinearity can be well described by the Duffing nonlinearity, and therefore, Eq. (1) is rewritten as

(B1)
$${\ddot{q}}_{1}+{\text{Γ}}_{1}{\dot{q}}_{1}+\omega_{1,\text{linear}}^{2}q_{1}+\alpha_{1}q_{1}^{3}=F_{2}q_{1}^{2}\cos\left(\omega_{2}t\right)\text{.}$$

It is convenient to write Eq. (B1) in the rotating frame with a reference of $\omega_{2}$/3,

(B2)
$$q_{1}=CQ\cos\left(\frac{\omega_{2}t}{3}\right)+CP\cos\left(\frac{\omega_{2}t}{3}\right),$$

where P and Q are two dimensionless quadratures and $C=\sqrt{8\omega_{1,\text{linear}}\delta \omega /3\alpha_{1}}$ with detuning $\delta \omega =\omega_{2}/3-\omega_{1,\text{linear}}$. In the rotating frame, the dimensionless quasienergy can be written as [24]

(B3)
$$g(Q,P)=\frac{1}{4}{\left(Q^{2}+P^{2}-1\right)}^{2}-f\left(Q^{3}-3P^{2}Q\right),$$

where $f=\left(F_{2}/3\sqrt{24\omega_{1,\text{linear}}\alpha_{1}\delta \omega }\right)$ is the dimensionless driving strength. The equation of motion for mode 1 is written as

(B4)
$$\dot{Q}=\partial_{P}g-\kappa Q,\;\dot{P}=-\partial_{Q}g-\kappa P,$$

where $\kappa =\left({\text{Γ}}_{1}/2\delta \omega \right)$. Trajectories in the phase diagram, as shown in Figs. 1(e) and 1(f), are obtained by numerically integrating Eq. (B4) from different initial conditions. The separatrices of the phase diagram are further calculated by doing gradient ascendant from the saddle points of g(Q,P) based on Eq. (B4) [27] (see Supplemental Material Note 1 [26]).

## APPENDIX C: MEASUREMENT SETUP

In this work, we use a well-established doubly clamped beam micromechanical resonator as the experimental platform to study the energy-decay processes of two nonlinearly coupled modes. The silicon resonator consists of a beam with lateral comb-drive electrodes for electrical actuation and detection [11,21]. In addition to the electrical measurement, we also perform optical measurement on the resonator out-of-plane motion as a separate measurement channel. Figure 7 shows a detailed schematic of the measurement setup. It is noteworthy to mention that the capacitive signal has a larger readout gain for the in-plane displacement since it is measured as the capacitive variance between the beam and the lateral electrode. On the contrary, the optical measurement, which is more sensitive to the out-of-plane displacement, is performed by a laser Doppler vibrometer with a helium-neon laser (λ = 633 nm) and maximum vertical detection limit of ±75 nm_._

During the experiment, the doubly clamped beam is placed in a vacuum chamber of the pressure of approximately 0.1 Pa (10^−3^ Torr), at room temperature. Two ac drives of independently set frequency are applied on the side electrodes with a dc bias of 6 V. The capacitive (via a low-noise current amplifier) and the optical readout are simultaneously monitored with a lock-in amplifier and an oscilloscope. For the ringdown experiment, we switch off the drives and monitor the motion of the beam from the two readout channels of the oscilloscope whose bandwidth is set to be much larger than the relaxation rates of the modes.

The experimental setup developed in this study advances the conventional setups shown in previous works [12], giving us additional control and detection ability. On the one hand, it allows us to independently drive the modes of interest by combining electrostatic forces of different frequencies from the lateral electrodes in an open-loop configuration, making it possible to independently set the arbitrary desired initial condition of the modes. On the other hand, the two channels of detection enable us to detect the displacement of in-plane and out-of-plane modes capacitively and optically, respectively, for achieving the best readout gain and measurement sensitivity. With the appropriate calibration, it allows us to accurately measure the total energy of the coupled-mode system which was not achievable before [11,12].

## APPENDIX D: DYNAMICS OF MODE 1 IN THE PTS PSEUDOPOTENTIAL AND INITIAL-PHASEDEPENDENT TRAJECTORY

The dynamics of mode 1 in the PTS pseudopotentials are shown in Fig. 8. Figure 8(a) presents in the phase diagram, four groups of experimental data obtained in the locking and bypassing experiment (Fig. 3). Three groups lock to the PTS basins around distinct stationary points, while one group (black) bypasses the PTS and directly decays to the ground state. Because of the strong Duffing nonlinearity, there is a rapid frequency change with amplitude, squeezing the basin in the radial direction. Therefore, we replot the same data in Fig. 8(b), enlarging the data at R>0.033 [labeled by the red dashed line in Fig. 8(a)] by redefining the radial coordinate as $Q^{\prime }+iP^{\prime }=(R-0.033)e^{i\varphi }$. The trajectory of ringdown is consistent with our schematic figure in Fig. 1(e), clearly demonstrating the locking and bypassing trajectory in the PTS pseudopotentials, and the precession (Ω) and decay (κ) of mode 1, as is also seen in Video 1 of the Supplemental Material [26].

To illustrate the initial phase dependence of the phase locking and bypassing, in Fig. 8(c), we present the 44 series of data from all those experiments where locking is observed out of the 100 total repeat experiments starting from random, uniformly distributed phases. The conditions correspond to the $A_{2,0}$ = 0.03 data in Fig. 4(b) (the set with locking probability approximately equal to 50%). We focus on the data just before entering the basin regions, by further enlarging and setting origin at R=0.040, i.e., $Q^{\prime \prime }+iP^{\prime \prime }=(R-0.040)e^{i\varphi }$, and truncating the data traces to include only R>0.042 [red dashed line in Fig. 8(b)]. The 44 groups of locking data are colored based on which basin they end up with. The remaining 56 groups of bypassing data, not shown here, concentrate within the white spaces between the discrete colored spirals, uniformly filling the phase space due to the uniform distribution of the initial relative phase.

Only initial conditions within the basin of attraction for each PTS stationary point (areas covered by the locking data points) lead to a locked state, while other initial conditions (white empty areas) result in bypassing to the ground state, consistent with the illustration in Figs. 1(e), 4(c), and 4(d). In the paper, we use the schematic generated with smaller frequency detuning to more clearly illustrate all the features on a single radial scale (no need to enlarge) to facilitate understanding.

## APPENDIX E: MECHANICAL SPECTRUM OF THE DEVICE

We drive and measure the mechanical spectrum of the resonator up to 1 MHz (approximately 15 times the fundamental frequency) shown as the blue dots in the upper panel of Fig. 9. The gray dots are the measurement noise floor measured at the stationary reference substrate. In addition, we numerically simulate the eigenfrequency of the devices and quantitatively match all the modeled mechanical modes of the system shown as the lower panel of Fig. 9, eliminating the possibility of hidden modes. The dashed line labels $n\times \omega_{\text{IR}}$ where $\omega_{\text{IR}}=\omega_{2,\text{linear}}/3$ is the internal resonance frequency and n=1,2,3,… Except for mode 1 and mode 3 (labeled red), there is no other commensurate frequency matching. As we describe in the paper, mode 1’s linear eigenfrequency is detuned lower than $\omega_{\text{IR}}$, but it matches $\omega_{\text{IR}}$ at a specific amplitude due to its Duffing-nonlinearity frequency shift.

## APPENDIX F: UNCERTAINTY ANALYSIS

There are two groups of data (blue squares and green diamonds) shown in Fig. 4(a), distinguished by how their initial conditions are prepared. Their uncertainties represented by the error bars in Fig. 4(a) are obtained separately for the two data types via slightly different methods.

For the case where mode 1 is locked to mode 2 initially (blue squares), the coherence time $t_{c}$ is defined as shown in Fig. 2(e), where the start of the coherence time is well defined as the time we turn off the drive (t=0). The end of the coherence time is defined as the point of unlocking and is determined with a larger uncertainty due to the oscillation of frequency. This is the leading uncertainty in $t_{c}$ for these data. To quantify it, we use one period of oscillation at unlocking, shown as positive and negative error bars (y axis) for each data point. The error bar of $A_{2,0}$ (x axis) indicates 1 standard deviation due to the detection noise of the $A_{2}$ measurement at the beginning of the ringdown (when the drive is turned off). The measurement noise is constant, and the differences in the oscillation frequency are small; thus, the data points share approximately the same horizontal and vertical uncertainties, as indicated by the black error bars in Fig. 4(a).

For the case where mode 1 is not locked to mode 2 initially (green diamonds), the coherence time is defined as indicated in Fig. 3(b), where both the start and the end of locking are uncertain due to the frequency oscillation. The uncertainty of $t_{c}$ is defined as an average of the oscillation periods at the start and the end of locking. Regarding the uncertainty of $A_{2,0}$, it is a combination of two statistically independent contributions. The first part is from the detection noise of $A_{2}$, the same as above. The second part is arising from the uncertainty at the starting point of the coherence period (locking time) due to the oscillation of frequency, as shown in Fig. 2(b). We define the peak-to-valley difference of $A_{2}$ during the first frequency oscillation period after locking as the second part of the uncertainty. We combine these two parts of uncertainty for $A_{2,0}$ via root-mean-square and depict as the error bars in the x axis for the green dots. The oscillation of frequency is initially relatively large, and the amplitude and frequency vary among the data; therefore, we characterize the uncertainties separately for points within each group of measurements shown as the red error bars on top of the corresponding groups of green diamond data points shown in Fig. 4(a).

## APPENDIX G: ENERGY TRANSFER BETWEEN MODES AND CALIBRATION

Since the time-averaged interaction energy is neglected, the conservation of energy during the coherence time equals the energy lost by mode 2 to the energy gained by mode 1 after accounting for their intrinsic losses. It allows us to simultaneously fit both mode amplitudes, calibrate them, and plot them on a common energy scale.

After turning off the external driving force, the amplitude of mode 1 remains constant or even increases for a finite period of time, and the energy of mode 2 decays much faster than its expected exponential relaxation via linear damping. The equations describing the amplitude of each mode during ringdown for a coupled two-mode system at internal resonance are

(G1)
$$\frac{dE_{1}}{dt}=-{\text{Γ}}_{1}E_{1}+W,\;\frac{dE_{2}}{dt}=-{\text{Γ}}_{2}E_{2}-W,$$

where $E_{1}=c_{1}A_{1}^{2}$ and $E_{2}=c_{2}A_{2}^{2}$ are the energies of mode 1 and mode 2, which are proportional to their measured amplitude square with proportionality constants of $c_{1}$ and $c_{2}$, respectively, and W is the energy exchange rate between the two modes, which is approximately constant in our system, since $\left(dE_{1}/dt\right)\approx 0$ during the coherence time.

By fitting the ringdown amplitude of the two modes with Eq. (G1), we obtain the calibration constant $\eta =[\left(W/c_{1}\right)/\left(W/c_{2}\right)]=\left(c_{2}/c_{1}\right)$, which is used for unifying the unit of energy of mode 1 and mode 2.

By solving Eq. (G1), we obtain the time-dependent amplitudes of mode 1 and mode 2:

(G2)
$$A_{1}^{2}=A_{1,0}^{2}e^{-{\text{Γ}}_{1}t}+\frac{W}{c_{1}{\text{Γ}}_{1}}\left(1-e^{-{\text{Γ}}_{1}t}\right),$$

$$A_{2}^{2}=A_{2,0}^{2}e^{-{\text{Γ}}_{2}t}-\frac{W}{c_{2}{\text{Γ}}_{2}}\left(1-e^{-{\text{Γ}}_{2}t}\right),$$

where $A_{1,0}$ and $A_{2,0}$ are the initial amplitudes of mode 1 and mode 2 at the beginning of locking, respectively. These equations are used to fit the data of Figs. 2(a) and 3(e) with $W/c_{2}$ as the only fitting parameter (black lines in the figure). Without loss of generality, the energy units are chosen such that $W/c_{1}=1$. After unlocking, the two modes dissipate energy at the rate given by their intrinsic dissipation rate ${\text{Γ}}_{1}$ and ${\text{Γ}}_{2}$, as expected. The blue and red dashed lines in Fig. 2(a) indicate the independent exponential decays of the modes ($E_{1}=E_{1,0}e^{-{\text{Γ}}_{1}t}$, $E_{2}=E_{2,0}e^{-{\text{Γ}}_{2}t}$).

As mode 2 is regarded as the driving force to mode 1 to support the PTS, we can solve Eq. (G2) to get a coherence time $t_{c}$ by considering the ringdown time of mode 2 from its initial amplitude to the threshold of $A_{2,\text{threshold}}\left(f_{0}\right)\approx 0.006\;\text{V}$ as measured in Fig. 4(a). With known ${\text{Γ}}_{2}$, $W/c_{2}$, and $A_{2,0}$, the coherence time can be written as

(G3)
$$t_{c}=-\frac{1}{{\text{Γ}}_{2}}\ln\left[\frac{A_{2,\text{threshold}}^{2}+{\text{Γ}}_{2}W/c_{2}}{A_{2,0}^{2}+{\text{Γ}}_{2}W/c_{2}}\right].$$

## APPENDIX H: THE FULLY COUPLED MODEL OF TWO NONLINEARLY COUPLED OSCILLATORS AT THE 1∶3 INTERNAL RESONANCE AND HOW IT IS REDUCED TO A PTS-LIKE MODEL

The free-ringdown system of two coupled Duffing oscillators at the 1∶3 internal resonance can be described by two equations of motion as

(H1)
$${\ddot{q}}_{1}+{\text{Γ}}_{1}{\dot{q}}_{1}+\omega_{1,\text{linear}}^{2}q_{1}+\alpha_{1}q_{1}^{3}+m_{1}^{-1}\partial H_{\text{int}}/\partial q_{1}=0\;\text{,}$$

(H2)
$${\ddot{q}}_{2}+{\text{Γ}}_{2}{\dot{q}}_{2}+\omega_{2,\text{linear}}^{2}q_{2}+\alpha_{2}q_{2}^{3}+m_{2}^{-1}\partial H_{\text{int}}/\partial q_{2}=0$$

with an interaction energy term for 1∶3 internal resonance and the values of coupling constant g, modal masses $m_{1}$ and $m_{2}$, and coefficients $\alpha_{1}$ and $\alpha_{2}$ dependent on the choice of the scales of the generalized coordinates $q_{1}$ and $q_{2}$. Taking the experimentally measured voltage values directly as the generalized coordinates, the Duffing coefficients of the two modes obtained from their measured Duffing spectrum are $\alpha_{1}\approx 2.41\times 10^{8}{\text{s}}^{-2}{\text{V}}^{-2}$ and $\alpha_{2}\approx -1.03\times 10^{7}{\text{s}}^{-2}{\text{V}}^{-2}$.

By taking the derivatives and defining coupling coefficients $g_{12}=\left(\text{g}/{\text{m}}_{1}\right)$ and $g_{21}=\left(\text{g}/{\text{m}}_{2}\right)$, we can write the equations of motion as

(H3)
$${\ddot{q}}_{1}+{\text{Γ}}_{1}{\dot{q}}_{1}+\omega_{1,\text{linear}}^{2}q_{1}+\alpha_{1}q_{1}^{3}+3g_{12}q_{1}^{2}q_{2}=0\;\text{,}$$

(H4)
$${\ddot{q}}_{2}+{\text{Γ}}_{2}{\dot{q}}_{2}+\omega_{2,\text{linear}}^{2}q_{2}+\alpha_{2}q_{2}^{3}+g_{21}q_{1}^{3}=0$$

when no external forces are applied.

It is instructive to consider the system in the rotating-wave approximation $q_{1}=u_{1}e^{\left(i\omega_{2,\text{,inear}}t/3\right)}+u_{1}^{*}e^{-\left(i\omega_{2,\text{linear}}t/3\right)}$ and $q_{2}=u_{2}e^{i\omega_{2,\text{linear}}t}+u_{2}^{*}e^{-i\omega_{2,\text{linarar}}t}$, and neglecting non-resonant rapidly varying terms:

(H5)
$${\dot{u}}_{1}+\frac{{\text{Γ}}_{1}}{2}u_{1}+i\left(\frac{\omega_{2,\text{linear}}}{3}-\omega_{1,\text{linear}}\right)u_{1}-i\frac{3\alpha_{11}}{2\omega_{1,\text{linear}}}{\left|u_{1}\right|}^{2}u_{1}$$

$$-i\frac{3g_{12}}{\omega_{1,\text{linear}}}u_{1}^{*2}u_{2}=0\;\text{,}$$

(H6)
$${\dot{u}}_{2}+\frac{{\text{Γ}}_{2}}{2}u_{2}-i\frac{3\alpha_{22}}{2\omega_{2,\text{linear}}}{\left|u_{2}\right|}^{2}u_{2}-i\frac{g_{21}}{\omega_{2,\text{linear}}}u_{1}^{3}=0.$$

With the separately measured parameters ${\text{Γ}}_{1}$, ${\text{Γ}}_{2}$, $\omega_{1,\text{linear}}$, $\omega_{2,\text{linear}}$, $\alpha_{1}$, and $\alpha_{2}$, we can fit the results using only two fitting parameters $g_{12}$ and $g_{21}$. For example, data in Fig. 2(a) can be fit by this complete model shown as Fig. 10 where $g_{12}\approx 3.32\times 10^{5}{\text{s}}^{-2}{\text{V}}^{-2}$ and $g_{21}\approx 2.98\times 10^{6}{\text{s}}^{-2}{\text{V}}^{-2}$ are fitted by achieving the least residual between the fit and the amplitude data.

To compare with experiment, we choose to use the experimental voltages directly as the generalized coordinates. This choice results in the unequal modal masses $m_{1}$ and $m_{2}$ for the two modes, and therefore unequal values of $g_{12}=\left(\text{g}/{\text{m}}_{1}\right)$ and $g_{21}=\left(\text{g}/{\text{m}}_{2}\right)$. For the theoretical analysis, it is conventional to choose the generalized coordinates ${\tilde{q}}_{1}$ and ${\tilde{q}}_{2}$ such that ${\tilde{m}}_{1}={\tilde{m}}_{2}$ and therefore, ${\tilde{g}}_{12}={\tilde{g}}_{21}$. This can be easily done by rescaling the generalized coordinate for mode 2 as ${\tilde{q}}_{2}=q_{2}\sqrt{\left(m_{2}/m_{1}\right)}=q_{2}\sqrt{\left(g_{12}/g_{21}\right)}\approx 0.33q_{2}$, obtaining

(H7)
$${\ddot{q}}_{1}+{\text{Γ}}_{1}{\dot{q}}_{1}+\omega_{1,\text{linear}}^{2}q_{1}+\alpha_{1}q_{1}^{3}+3\tilde{g}q_{1}^{2}{\tilde{q}}_{2}=0\;\text{,}$$

(H8)
$${\ddot{\tilde{q}}}_{2}+{\text{Γ}}_{2}{\dot{\tilde{q}}}_{2}+\omega_{2,\text{linear}}^{2}{\tilde{q}}_{2}+{\tilde{\alpha }}_{2}{\tilde{q}}_{2}^{3}+\tilde{g}q_{1}^{3}=0$$

with the rescaled Duffing coefficient ${\tilde{\alpha }}_{2}=\alpha_{2}\left(m_{1}/m_{2}\right)\approx 9.0\alpha_{2}$ and a common rescaled coupling coefficient $\tilde{g}=\sqrt{g_{12}g_{21}}$. We note that the interaction energy $H_{\text{int}}$, kinetic energies $\frac{1}{2}m_{1}{\dot{q}}_{1}^{2}$ and $\frac{1}{2}m_{2}{\dot{q}}_{2}^{2}$, and potential energies $\frac{1}{2}m_{1}\omega_{1,\text{linear}}^{2}q_{1}^{2}$ and $\frac{1}{2}m_{2}\omega_{2,\text{linear}}^{2}q_{2}^{2}$ are the coordinate-scale-invariant physical quantities and remain unchanged. The energy and phase results presented in Fig. 10 are also invariant with respect to coordinate scaling.

Another option is to use the calibration constant obtained from Appendix G which is based on the assumption of constant energy flow W. The calibration constant in Eq.(G1) $\sqrt{c1/c2}=\left(m_{1}/m_{2}\right)$ is calibrated to be approximately equal to 8.8 and used to put the mode energies on the common energy scale in Figs. 2 and 3. By fixing the ratio $\left(m_{1}/m_{2}\right)=8.8$, the two fitting parameters in Eqs. (H3) and (H4) can be reduced to a single adjustable parameter $g_{12}$, where $g_{21}=8.8\;g_{12}$. Fitting it to the experiment results, as shown in Fig. 11, yields $g_{12}\approx 3.07\times 10^{5}{\text{s}}^{-2}{\text{V}}^{-2}$ and $g_{21}=8.8g_{12}\approx 2.70\times 10^{6}{\text{s}}^{-2}{\text{V}}^{-2}$. Naturally, this single-parameter fit gives nearly the same results as that of the complete two-fitting parameter fit ($g_{12}\approx 3.32\times 10^{5}{\text{s}}^{-2}{\text{V}}^{-2}$ and $g_{21}\approx 2.98\times 10^{6}{\text{s}}^{-2}{\text{V}}^{-2}$).

Equation (H6) can be rewritten as

(H9)
$${\dot{u}}_{2}+\left[\frac{{\text{Γ}}_{2}}{2}+Im\left(\frac{g_{21}}{\omega_{2,\text{linear}}}\frac{u_{1}^{3}}{u_{2}}\right)\right]u_{2}$$

−i[3α222ω2,linear|u2|2+Re(g21ω2,linearu13u2)]u2=0

to explicitly express the interaction term as a change in the damping rate $\text{Im}\left[\left(g_{21}/\omega_{2,\text{linear}}\right)\left(u_{1}^{3}/u_{2}\right)\right]$ and the frequency Re[(g21/ω2,linear)(u13/u2)].

Experimentally, we observe that mode 2 frequency and amplitude change gradually, within the experimental resolution, in all regimes except very near the unlock time. This suggests considering Eq. (H9) in the limit where the rate term $\left(g_{21}/\omega_{2,\text{linear}}\right)\left(u_{1}^{3}/u_{2}\right)$ is small. In this limit, mode 2 oscillates at the frequency dictated by its linear frequency and Duffing-nonlinearity backbone, regardless of the dynamics of mode 1 [seen in Figs. 2(d) and 3(b) as red dots]; i.e., mode 1 dynamics barely affect the frequency and phase of mode 2. Specifically, the phase of mode 2 oscillation becomes fully determined by the Duffing nonlinearity and changes slowly with the decreasing mode 2 amplitude, on the timescale determined by the damping rates of the system. This allows considering Eq. (1) for mode 1 with interaction force of slowly changing amplitude and frequency, and applying the insights that have been previously developed for a single-mode period-tripling state.

Within this single-mode PTS description, near one of three stationary lock points, a high-Q mode (e.g., mode 1) undergoes precession in the local pseudopotential at a frequency Ω, which defines the fastest dynamical timescale. The approximate PTS-like description is applicable when the force changes slowly on this timescale; i.e., both the PTS stationary point location and the pseudopotential profile change adiabatically. Qualitatively, the PTS description is self-consistent when the rate term $\left(g_{21}/\omega_{2,\text{linear}}\right)\left(u_{1}^{3}/u_{2}\right)$ describing the modification to mode 2 dynamics by mode 1 is much smaller than Ω.

While mode 2 frequency is barely affected by mode 1, its energy-loss rate to mode 1 $2\text{Im}\left[\left(g_{21}/\omega_{2,\text{linear}}\right)\left(u_{1}^{3}/u_{2}\right)\right]$ can be comparable to its weak intrinsic damping ${\text{Γ}}_{2}$. Therefore, we retain this part of the interaction, which leads to the energy flow W between modes in Eq. (G1), and increases the overall mode 2 loss rate, as seen in Figs. 2(a) red and 3(e) red dots.

To summarize, at the above analytically discussed and experimentally demonstrated limit, the major dynamics of mode 2 (including phase and amplitude) in the rotating frame are on the timescale of approximately $1/{\text{Γ}}_{1,2}$. Given the low energy-loss rate $1/{\text{Γ}}_{1,2}$, mode 2 acts as an adiabatically varying nonlinear drive to mode 1. Mode 1 dynamics can be described using the PTS picture with a slowly varying period-three force at the frequency of $\omega_{2}\approx 3\omega_{1}$. Specifically, from the perspective of mode 1 at this limit, mode 2 provides a period-three harmonic drive with slowly varying amplitude and frequency, i.e., $q_{2}(t)\propto F_{2}(t)\cos\left[\phi_{2}(t)\right]$, where ${\dot{\phi }}_{2}(t)=\omega_{2}$. As a result, Eq. (H3) is reduced to Eq. (1), where the coupling term becomes $q_{1}^{2}F_{2}(t)\cos\left[\phi_{2}(t)\right]$ from $3g_{12}2q_{1}^{2}q_{2}$.

The PTS model provides a valid description of our system, evident by the slowly evolving $F_{2}(t)$ and $\omega_{2}(t)$ (given by the measured mode 2 dynamics) relative to the fast frequency and the small-amplitude oscillations of the locked mode 1, and frequency-amplitude relationship defined by Duffing backbones. However, as shown by the numerical modeling using the fully coupled model in Fig. 12, the locking phenomena can even occur with parameters where the PTS model does not strictly apply. A general approach can be used to explore the broader parameter range where the discovered phase-locking phenomenon can occur. Here we illustrate the generality of the observed phenomena by numerically simulating the fully coupled model with coupling strength, Duffing nonlinearities, and damping rates varied within 2 orders of magnitude. As shown in Fig. 12, the phase-locking state remains there, showing this physical behavior is present across a wide range of multiple system parameters. In Figs. 12(b), 12(c), 12(e), 12(g), and 12(j), although the condition of PTS locking by a force with a smoothly varying amplitude and frequency already breaks down, evident by the oscillating mode 2 amplitude, the phase-locked state still exists, evident by the sustained constant-time-averaged mode 1 amplitude and constant relative phase.

The description of our system motion can be further simplified from the complete model when an additional condition is valid, namely, that mode 1 also approximately follows its Duffing backbone; i.e., the modification of mode 1 frequency-amplitude relation by the interaction term is small. This condition is valid in our system since the measured frequency-amplitude relation in Figs. 2 and. 3 is consistent with its Duffing backbone. This condition means that the time-averaged interaction energy is negligible compared to either of the mode energies, including in the locked PTS state. As a result, the extra energy loss from mode 2 in addition to natural damping equals the extra energy gain of mode 1, as described by Eq. (G1). Meanwhile, the phase-locked-mode amplitudes are fully determined by their common frequency at all times. The time evolution of the amplitudes is determined by the intrinsic energy-loss rates decreasing the total energy of the system, while the energy flow between the modes is such that their individual amplitudes remain on their backbones at some common frequency changing in time. In particular, when the mode 2 backbone is linear (near vertical), the mode 1 time-averaged frequency must remain constant to maintain the lock, which results in the mode 1 time-averaged amplitude remaining constant during lock as shown in Fig. 2(a). Equation (G1) with a single parameter, W, achieves good agreement for the energy time evolution in Figs. 2 and 3, and the coherent time fit in Fig. 4 [Eq. (G3)].

While this energy-transfer view is particularly compelling in its simplicity, it is not a necessary condition for the PTS description of the mode 1 evolution. The only benefit of this condition in our work is providing a simple and intuitive way to quantitatively fit the slow energy evolution of both modes and the coherent time, while neglecting the faster features of the full PTS model dynamics from mode 1, such as the small but observable oscillations of mode 1 phase and amplitude at the precession frequency Ω.

In summary, while we numerically confirm that the system can be modeled well with fully coupled equations, the reduced model advances the physical understanding of the mode interaction process, while providing a good quantitative description. The reduced model makes a connection between fully coupled modes and the PTS for the lower mode, which intuitively explains the novel dynamics observed in this work: initial-phase-dependent bypassing or phase locking at 2π/3 relative phase increment; frequency and amplitude evolution, including the nonmonotonic lower-mode energy evolution; long coherent time and locking probability as a function of the initial conditions.
