# Supplementary material for: Persistent Nonlinear Phase-Locking and Nonmonotonic Energy Dissipation in Micromechanical Resonators
Source: Phys Rev X. Author manuscript; Available in PMC 2024 Apr 26. (PMC11047221; doi:10.1103/physrevx.12.041025)
Supplement: Supp1 [file NIHMS1858147-supplement-Supp1.pdf]

Supplementary Information

Persistent nonlinear phase-locking and non-monotonic energy dissipation in micromechanical resonators

Mingkang Wang[1,2], Diego J. Perez-Morelo[1,2], Daniel Lopez[3,1], and Vladimir A. Aksyuk[1]
[1]Microsystems and Nanotechnology Division, National Institute of Standards and Technology, Gaithersburg, MD 20899 USA
[2]Institute for Research in Electronics and Applied Physics, University of Maryland, College Park, MD 20742, USA
[3]Materials Research Institute, Penn State University, University Park, PA 16802

S1- Simulation of Period tripling states

As shown in Eq. (1), Mode 1 performs as an anharmonic oscillator with resonance frequency dependent on amplitude, and it is subject to the external excitation from Mode 2 via the nonlinear coupling term $\propto q_1^2 q_2$. In this specific experiment, the amplitude-frequency relationship for Mode 1 is well described by the Duffing form, therefore, Eq. (1) is rewritten as [1-3]:

$$\ddot{q}_1 + \Gamma_1 \dot{q}_1 + \omega_{1,linear}^2 q_1 + \alpha_1 {q_1}^3 = F_2 q_1^2 \cos(\omega_2 t) \qquad (S1)$$

where α_1 stands for the Duffing coefficient and $\omega_2 \approx 3\omega_1$ where $\omega_1^2(q_1) = \omega_{1,linear}^2 + \alpha_1 q_1^2$ with detuning $\delta\omega = \omega_2/3 - \omega_{1,linear}$. The product of the $\cos(\omega_2 t)$ term and the approximately-harmonic displacement term ${q_1}^2 \propto \cos(\omega_2 t/3)^2$ provides a driving force with a harmonic component at the frequency $\omega_2/3 = \omega_{1,linear} + \delta\omega$ to drive Mode 1.

Following Ref [4], it is convenient to write Eq. (S1) in the rotating frame with a reference frequency of $\omega_2/3$

$$q_1 = CQ\cos\left(\frac{\omega_2 t}{3}\right) + CP\cos\left(\frac{\omega_2 t}{3}\right) \qquad (S2)$$

where P and Q are two dimensionless quadratures and $C = \sqrt{\frac{8\omega_{1,linear}\delta\omega}{3\alpha_1}}$. In the rotating frame, the dimensionless quasienergy can be written as:

$$g(Q,P) = \frac{1}{4}(Q^2 + P^2 - 1)^2 - f(Q^3 - 3P^2 Q) \qquad (S3)$$

where $f = \frac{F_2}{3\sqrt{24\omega_{1,linear}\alpha_1\delta\omega}}$ is the dimensionless driving strength. The equation of motion for Mode 1 is written as:

$$\dot{Q} = \partial_P g - \kappa Q$$
$$\dot{P} = -\partial_Q g - \kappa P \qquad (S4)$$

where $\kappa = \frac{\Gamma_1}{2\delta\omega}$. Trajectories in the phase diagram [as shown in Fig. 1e] can be obtained by numerically integrating (S4) from different initial conditions.

By solving for $\dot{Q} = 0$ and $\dot{P} = 0$, we obtain 7 solutions. Then we use $\ddot{Q} < 0$ and $\ddot{P} < 0$ to select the 3 stable states (red dots in separatrix plot), 3 saddle points, and 1 trivial-state.

The separatrix in the *P*-*Q* phase diagram is on the ridge of $g(P,Q)$ which separates the phase diagram into 4 regimes. To obtain the separatrix, we set the initial condition *P*(0), *Q*(0) at around the saddle points which are on the minimum point of a ridge of $g(P,Q)$, and do gradient ascendant

of P, Q , i.e. inverting time in (S4) and numerically integrating, as:

$$Q(t+dt) = Q(t) - (\partial_P g - \kappa Q)dt$$
$$P(t+dt) = P(t) - \left(-\partial_Q g - \kappa P\right)dt. \qquad (S5)$$

The trajectory of P(t)-Q(t) is the separatrix.

Note, the present separatrixes in the paper have $\kappa = 0.1$, while our real system losses are much smaller, $\kappa < 0.001$. We use bigger κ for visual clarity of the diagrams presented in our paper, to avoid the helix of separatrix winding too dense and narrow. The performance of the system is qualitatively the same for different κ. In order to show the separatrix under different driving strength, we set medium $f = 0.0800$ for Fig. 1(e) and the red line in Fig. 2(f), and small $f = 0.0034$ for Fig. 1(f) and the blue line in Fig. 2(f). Large $f = 0.25$ and medium $f = 0.05$ are used for Fig. 4 (c) and (d), respectively.

To illustrate the evolution of an oscillator under a slowly time-varying driving strength (mimicking the gradual amplitude decay of Mode 2), we generate a movie (Supplementary Movie 1) where the oscillator is starting from two different initial conditions having the same amplitude but slightly different phases (<0.1 rad). For the Movie, $\kappa = 0.1$ is set as a constant while f linearly changes from 0.25 to 0 during the ringdown. In the beginning, the oscillator freely decays. The red dot depicts the trajectory of the oscillator with an initial phase that allows it to lock into the PTS, while the blue one shows the trajectory that bypasses the PTS and decays to the trivial state directly. At the time when the gradually decreasing f reaches a threshold $f_0 \approx 0.033$, the PTS attractors disappear. The oscillator (red dot) unlocks from the PTS and rings down to the trivial state. The simple PTS model qualitatively presents the dynamics of Mode 1 observed in Figure 2.

The same dynamics are also depicted in Figure S1, presenting the amplitude as a function of time during the decay from the two initial conditions, numerically integrating (S4) with f gradually decreased in time, as noted above. The sharp amplitude drop visible when the resonance is being bypassed (blue) is consistent with the measurement in Fig. 3c. The linear-damping relaxation rate is recovered once the modes are unlocked. Note, here the frequency ω_2 is maintained constant while f is decreasing, therefore, the locked state (red) shows a constant amplitude. This is consistent with the measurements in Fig. 2a, where Mode 2's frequency is nearly unchanged (Fig. 2d). The dynamics of the PTS under time-varying modulation frequency and force is more fully considered in the next Supplemental section, showing Mode 1 exhibiting a non-monotonic energy change.

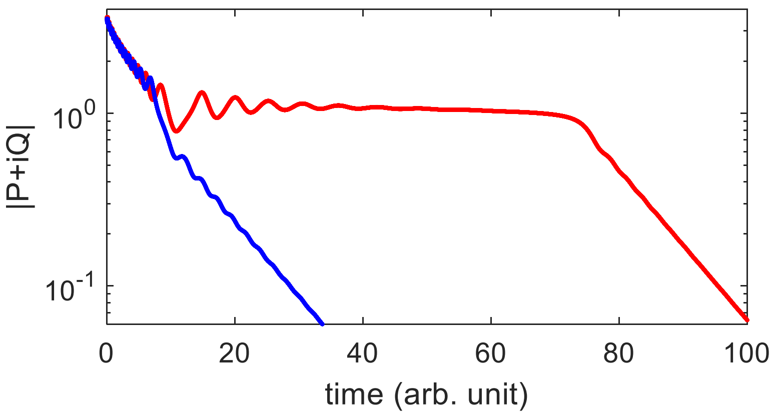

Figure S1 Amplitude of the oscillators shown in Supplementary Movie 1. The red and blue lines correspond to the dots in Supplementary Movie 1 with the corresponding colors.

S2- PTS under dynamically varying parameters

As we discussed in the previous section, Mode 1 can be regarded as an oscillator subject to PTS, governed by the Eq. (S1), where Mode 2 provides the external drive with frequency $\omega_2 \approx 3\omega_1$ and force $F_2 \propto q_2$, whenever any fast dynamics of Mode 2 due to the coupling can be neglected. This simple model not only can qualitatively explain the behavior of Mode 1 in this experiment, such as the locking and bypass trajectories [illustrated in Fig.1 (e), (f); Fig. S1], the locking probability [Fig 4(c), (d)] and its striking energy decay reversal. The model can also provide a common general approach for describing a multitude of different experimentally observed energy-dependent dissipation rates in systems at internal resonance [1-3], accounting for both an increased as well as decreased and even negative dissipation in the locked state.

Figure S1 uses the PTS model to explain how PTS locks an oscillator [Mode 1] and makes it persist without decay for a period of time (coherence time), longer than its intrinsic dissipation time, during which the driving force is gradually decreasing. It describes the behaviors of Mode 1 in Fig. 2(a) where Mode 2 does not show strong nonlinearity, i.e., the driving frequency is nearly constant $\omega_2 \approx \omega_{2,linear}$. In order to explain the non-monotonic dissipation rate showing in Fig. 3(c) by this PTS model, we need to let the driving frequency from Mode 2 ω_2 vary together with the force F_2, so that we can take into account the amplitude-dependent frequency of nonlinear Mode 2. As the reference frequency $\omega_2/3$ of the rotating frame is also changing in time in this case, we use the stationary frame to simulate the dynamics [i.e. directly numerically integrating Eq. S1] and extract the amplitude of the oscillation to avoid any additional approximations or ambiguity. However, we note that this is equivalent to slowly changing variables F_2 and $\omega_2/3$ in the rotating frame, which now rotates at a variable rate.

The equation of motion is shown as Eq. (S1) where $\omega_{1,\text{linear}}/2\pi = 10$ kHz, $\Gamma_1/2\pi = 0.1$ Hz (quality factor $= \omega_{1,\text{linear}}/\Gamma_1 = 1.0 \times 10^5$), $\alpha_1 = 1.0 \times 10^4 \text{ rad}^2\text{ s}^{-2}\text{ nm}^{-2}$ are used for this example. In our paper, Mode 2 has a negative Duffing nonlinearity, therefore, ω_2 increases following the gradual decrease of $q_2 \propto F_2$ to mimic the energy loss of nonlinear Mode 2. The blue lines in Figure S2(b),(c) shows the time-dependent F_2 and $\delta\omega$ that we used in the simulation where F_2 decreases from 1.0×10^5 rad^2 s^{-2} nm^{-1} to 0 rad^2 s^{-2} nm^{-1} between $t = 0.1$ s and $t = 0.33$ s and $\delta\omega/2\pi$ increases from 100 Hz to 150 Hz between $t = 0.1$ s and $t = 0.2$ s. In Fig. S2(a), the oscillator is locked to the stable PTS between $t = 0$ s and 0.1 s. As ω_2 increases, the oscillator exhibits an energy gain, qualitatively similar to the one observed in our experiment shown in Fig. 3(c). With decreasing F_2, it unlocks from PTS at $t \approx 0.2$ s where $F_2 < 0.6 \times 10^5$ rad^2 s^{-2} nm^{-1} and exponentially decays with its intrinsic dissipation rate afterward. The non-monotonic amplitude (energy) clearly indicates that the drive pumps energy into the oscillator. Intuitively, if we treat the period-3 drive as a perturbation, the oscillator (Mode 1) with a positive α_1 follows its hardening eigenfrequency-energy dependence, where the amplitude increases with the locked increasing frequency. The sweeping-up frequency of the drive (Mode 2) drags the phase-locked oscillator (Mode 1) to a higher frequency, leading to a larger amplitude (pumping energy into the oscillator, gain effect). This simple simulation qualitatively explains the novel non-monotonic dissipation rate observed in our experiment.

More generally, the PTS model can also explain the rapid energy loss effect for oscillators at internal resonance, as has been recently observed experimentally in another system [3]. In this case,

the nonlinear drive pumps energy out from the oscillator in the locked state. For this numerical experiment, the parameters of the oscillator remain the same, we only reverse the sign of $\delta\omega$, making ω_2 decrease with decreasing F_2, shown as the red lines in Fig. S2(b),(c). As a result, the oscillator decays faster than its intrinsic dissipation rate while locked in the PTS with time-varying driving parameters [between approximately 0.1 s and 0.2 s]. The faster decay rate compared to its intrinsic decay rate clearly shows that the time-varying drive pumps energy out of the oscillator locked to the PTS in this case.

It is noteworthy that the rapid energy gain/loss is mainly due to the time-varying detuning which drags the locked mode. The time-varying nonlinear force does not contribute much to the amplitude change. The black line in Fig. S2(a) shows the case where the detuning $\delta\omega/2\pi = 100\text{ Hz}$ is constant, while the driving force F_2 decreases following the same trend as before (black lines in Fig. S2(b),(c)). Here the amplitude of the mode does not show rapid changes. It shows a similar trend as Fig. S1 where the mode maintains a nearly constant amplitude during locking ($t \approx 0.1 - 0.2\text{ s}$) even though the force is decreasing. After unlocking at $t \approx 0.2\text{ s}$ where $F_2 < 0.6 \times 10^5$ rad^2 s^{-2} nm^{-1}, the mode begins to lose energy with its intrinsic loss rate.

Although the different behaviors at internal resonance shown in Ref. [2,3] (main text Ref. [11, 12]) and present work are also observed within our PTS model by simply changing the sign of Mode 2's Duffing coefficient, we do not have enough information on whether this explanation applies to Ref. [3].

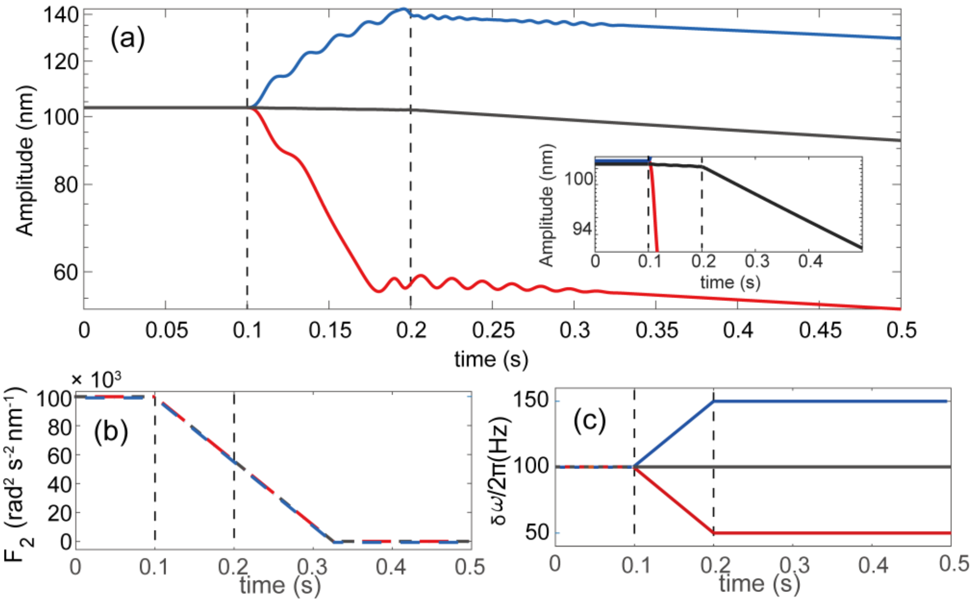

Figure S2 Numerically modeled dynamics of oscillators in PTS with time-varying parameters. $\omega_{1,linear}/2\pi = 10\text{ kHz}$, $\Gamma_1/2\pi = 0.1\text{ Hz}$, $\alpha_1 = 1.0 \times 10^4\ \text{nm}^2/\text{s}^2$ are fixed. F_2, $\delta\omega$ change as shown in (b), (c), resulting in the ringdown shown in (a) marked with the corresponding colors. The opposite tuning direction of driving frequency between $t = 0.1$ s and $t = 0.2$ s drags the locked mode to a higher/lower oscillation frequency, exhibiting rapid energy gain/loss faster than its intrinsic dissipation rate.

S3- Nonlinear coupled modes in resonance

We conjecture that the model we have presented may provide a useful approximate description for a broad range of systems near integer fraction resonances. Specifically, many such systems may exhibit stable locked states between two nonlinear coupled modes, whenever at least one of them is described by an amplitude-dependent eigenfrequency. The direction of the energy flow needed to maintain the locked state is determined by the modes' eigenfrequency-amplitude dependences (as illustrated in the numerical experiment in Section S4 and further discussed below). The stability of the locked state for different signs of these dependencies, as well as the conditions for applicability of the adiabatic assumption, are interesting subjects for future research, and may be studied further using existing general theoretical approaches [5, 6], however, the following consideration may serve as a useful guide.

In our simplified model, we make a few assumptions. We start by assuming the energy of the system is predominantly described by the sum of the energies of the two modes, neglecting the time-averaged energy of the interaction term. We further neglect the higher harmonics and describe each mode by its harmonic eigenfrequency being dependent on its energy. Duffing oscillator is just one specific example of such an energy-eigenfrequency relationship. Closely related is the assumption that the modes are constrained to their eigenfrequency-amplitute relationships, largely independent of the motion of the other mode: neglecting the time-averaged interaction energy makes the eigenfrequency-energy relationships for each mode independent of the energy of the other mode. These frequency-energy relationships $\omega_{k,osc}(E_k); k = 1,2$ are the first cornerstone of the proposed model.

Within this description, modes are allowed to exchange energy when their frequencies are commensurate. Energy conservation, after accounting for independent dissipation by each mode, is the second cornerstone of the model,

$$\frac{dE_1}{dt} = -\Gamma_1 E_1 + W(t) \qquad (S6)$$

$$\frac{dE_2}{dt} = -\Gamma_2 E_2 - W(t)$$

where $W(t)$ is the energy exchange rate (power flow) between the modes, which is time-dependent, generally. (Cf. Eq. 6, where power flow is near-constant for our specific experimental system).

The third cornerstone is the persistent phase-locked state on resonance, similar to the period tripling state for a single nonlinearly-driven Duffing oscillator, with the higher frequency mode acting as the nonlinear drive. The final key simplification here is the assumption that the energy, and therefore also the frequency, of the higher frequency mode is changing sufficiently slowly, such that the stable states of period tripling states driven by the higher frequency mode evolves adiabatically compare to the dynamics of lower frequency mode. This specifically assumes the interaction with Mode 1 does not result in significant dynamical changes to Mode 2 frequency and amplitude other than due to the gradual energy gain/loss of Eq. (S6). This assumption is valid under our specific experimental conditions, with only small deviations, i.e., the slight *amplitude* oscillations observed in the Mode 1 and Mode 2 data in Figure 3c,e, and a small frequency 'pull' near the unlocking point if Figure 2d. Importantly, the Mode 2 *phase* remains constant or varies very smoothly everywhere except near the unlocking point. The observed Mode 1 phase oscillations are expected for an oscillator settling into its PTS following our model. The exact general conditions

for the validity of this assumption remain to be investigated, such as by considering the perturbation from the small Mode 1 motion near the PTS on Mode 2, and the corresponding perturbation force back on Mode 1 using the more general coupled description [6].

The PTS-like locking to an external drive $F\cos(\omega_2 t)$ at frequency $\omega_2 \approx n\omega_1$ is not limited to a specific value of $n = 3$, but may exist for arbitrary integer $n \geq 1$, provided a nonlinear drive term is present, of the form $Fq_1^{n-1}\cos(\omega_2 t)$. Such effective drive term may arise from a nonlinear interaction or from a linear interaction with a nonlinear resonator mode (i.e., linear drive at the n-th harmonic). Here $n = 1$ is a simple linear drive on resonance, resulting in one non-zero state, and $n = 2$ is the common parametric drive at twice the frequency, resulting in two non-zero states, etc. As has been noted by others [5], the PTS at $n = 3$ is distinct from the lower n. For example, the stable trivial state co-exists with the PTS states over a frequency range.

The existence of such symmetry-breaking states for $n \geq 2$ is not limited specifically to Duffing nonlinearity, but rather is the property of any oscillator with an energy-dependent eigenfrequency $\omega_1(E_1)$ near the internal resonance, with the energy $E_1 = E_{1,res}$, where $E_{1,res}$ is defined by the period-n driving frequency ω_2, i.e. $\omega_1(E_{1,res}) = \omega_2/n$. When the drive is applied by a higher-order coupled mode (Mode 2), we have $\omega_1(E_{1,res}) = \omega_2(E_{2,res})/n$. Since we are interested in describing systems near internal resonances, we can linearize the $\omega_k(E_k); k = 1,2$:

$$\omega_k(E_k) = \omega_k(E_{k,res}) + (E_k - E_{k,res})\frac{d\omega_k}{dE_k}(E_{k,res}). \qquad (S7)$$

Since the PTS (n = 3) for a Duffing oscillator have been extensively studied, it is useful to reduce the motion of any nonlinear mode with $\frac{d\omega}{dE}(E_{res}) \neq 0$ sufficiently close to a PTS at $\omega_{res} = \omega(E_{res})$ to the equivalent Duffing oscillator having the same E_{res}, $\omega(E_{res})$ and $\frac{d\omega}{dE}(E_{res})$. Here we derive expression (S10) for the Duffing oscillator and explicitly provide the equivalent Duffing PTS parameters ω_0, α and $\delta\omega$ for a mode with given E_{res}, $\omega(E_{res})$ and $\frac{d\omega}{dE}(E_{res})$.

For a Duffing oscillator with a linear resonance frequency of ω_0 and a Duffing coefficient α, the eigenfrequency-energy relationship is given by $\omega^2 = \omega_0^2 + \alpha\langle q^2\rangle$ and $E = K\langle q^2\rangle$, accounting for both kinetic and potential energies, with a stiffness $K = m\omega_0^2$ for a modal mass m. To simplify, we assume the q, energy, and force are scaled such that $m = 1$. Assuming $\alpha\langle q^2\rangle \ll \omega_0^2$ we have $\omega(E) = \sqrt{\omega_0^2 + \frac{\alpha}{\omega_0^2}E} \approx \omega_0 + \frac{\alpha}{2\omega_0^3}E$, or, in the form of Eq (S10),

$$\omega(E) \approx \omega_0 + \delta\omega + \left(E - 2\frac{\omega_0^3}{\alpha}\delta\omega\right)\frac{\alpha}{2\omega_0^3}. \qquad (S8)$$

It is evident that for a nonlinear mode near a PTS with given E_{res}, $\omega(E_{res})$ and $\frac{d\omega}{dE}(E_{res})$, the equivalent Duffing PTS parameters are $\delta\omega = E_{res}\frac{d\omega}{dE}(E_{res})$, $\omega_0 = \omega(E_{res}) - E_{res}\frac{d\omega}{dE}(E_{res})$, and $\frac{\alpha}{2\omega_0^3} = \frac{d\omega}{dE}(E_{res})$. The equivalent Duffing oscillator loss rate remains the same as the nonlinear mode loss.

Now, we consider again our model where an adiabatically-varying higher-frequency mode drives the lower-frequency mode of energy-dependent eigenfrequency. In a simple case of linear

Mode 2, $\frac{d\omega_2}{dE_2} = 0$, we have the familiar PTS-like state with a constant-frequency nonlinear drive from Mode 2. Here we note that for Duffing Mode 1 changing a stiffening nonlinearity with a positive detuning to a softening nonlinearity with a negative detuning retains the same PTS but with a mirrored phase diagram in the $\omega_2/3$ rotating frame (Figs. 1e, f). More generally for modes of arbitrary energy-frequency relationship, the mirrored phase diagram will also present in the vicinity of the internal resonance upon changing $\frac{d\omega_1}{dE_1} \to -\frac{d\omega_1}{dE_1}$. In the rotating frame, near the resonance, the Mode 1 frequency-amplitude dependence is fully defined by

$$\omega_1(E_1) - \frac{\omega_2}{n} = \left(E_1 - E_{1,res}\right) \frac{d\omega_1}{dE_1} \tag{S9}$$

and the $\left|\frac{d\omega_{1,osc}}{dE_1}\right|$, together with the loss rate, describes Mode 1 dynamics near the internal resonance when dynamic backaction of Mode 1 on Mode 2 can be neglected.

Whenever the locked state $\omega_1(E_1) = \omega_2(E_2)/n$ is maintained in time, by taking a time derivative we obtain

$$\frac{dE_1}{dt}\frac{d\omega_1}{dE_1} = \frac{1}{n}\frac{dE_2}{dt}\frac{d\omega_2}{dE_2}. \tag{S10}$$

For known $\frac{d\omega_1}{dE_1}$ and $\frac{d\omega_2}{dE_2}$, Eq (S9) and (S13) can be solved to find the energy exchange rate $P(t)$, the time evolution of the system energies $E_k(t)$ and, therefore, the frequencies $\omega_k\left(E_k(t)\right)$. Eq. (S10), when combined with Eq. (S6), describes the system evolution in the locked state on the long timescale as it is ringing down, neglecting any dynamics near the locked state.

Understanding the exact fully-coupled dynamics on the short timescale near the locked state and the general conditions for the existence of the persistent locked state is beyond the scope of this study. However, the PTS-like locked state for $\frac{d\omega_2}{dE_2} \neq 0$ can be semi-quantitatively understood in the simplified limit, Mode 2 is still treated as the nearly-constant external drive for Mode 1, but now with Mode 2 frequency varying with changes in its energy as a function of time. Similar to previously considering Mode 1 in the rotating frame of a constant frequency ω_2/n, we now consider Mode 1 in the reference frame rotated at the variable frequency $\omega_2(E_2)/n$ defined by Mode 2. Neglect energy loss due to dissipation, $E_1 + E_2 = E_{1,res} + E_{2,res}$, and using Eq (S10) near a common resonance frequency we obtain

$$\omega_1(E_1) - \frac{\omega_2(E_2)}{n} = \left(E_1 - E_{1,res}\right)\frac{d\omega_1}{dE_1} - \frac{1}{n}\left(E_2 - E_{2,res}\right)\frac{d\omega_2}{dE_2} =$$

$$= \left(E_1 - E_{1,res}\right)\left(\frac{d\omega_1}{dE_1} + \frac{1}{n}\frac{d\omega_2}{dE_2}\right). \tag{S11}$$

Eq. (S11) has the same form as Eq. (S9) for the locked state dynamics with linear Mode 2, except for having an *effective* Mode 1 frequency-energy dependence

$$\left(\frac{d\omega_1}{dE_1}\right)_{effective} = \frac{d\omega_1}{dE_1} + \frac{1}{n}\frac{d\omega_2}{dE_2}. \tag{S12}$$

Therefore, Mode 1 dynamics near the locked state with both modes nonlinear may be quantitatively described similarly to the case with linear Mode 2, but using an effective nonlinearity for Mode 1.. However, we admit that a more consistent treatment of the dissipation for each mode, as well as a

consistent fully coupled treatment of the system in general, has to be applied for a more accurate description. The analysis provided in this section is only intended to illustrate how the two-mode PTS-like locked state model we developed to describe our experiments might be generalized to include higher integer frequency ratio n as well as an arbitrary eigenfrequency-energy dependencies $\omega_k(E_k)$ for each of the two nonlinear modes. In conjunction with Supplemental Section 4, this illustrates how, depending on each Mode's nonlinearities, the persistent locked state leads to either an increase or a decrease in the apparent observed decay rate of a given mode.

When appropriate nonlinear coupling terms exist, multiple modes and applied drive stimuli can resonantly exchange energy when the frequency matching conditions of the form $\sum m_k f_k = 0$ are satisfied for some integer m_k. Here the external stimuli are mathematically equivalent to infinitely stiff and massive lossless modes. Since the lowest-order nonlinear terms not forbidden by symmetry are often dominant, the coupling is strongest for the lowest number of participating modes and smallest integers. Such coupled nonlinear systems are commonly encountered and studied, including photonic, nanomechanical, cavity optomechanical, RF and other domains, where nonlinear signal generation and transduction are of high interest. These systems are less constrained and general conditions leading to the persistent locked states are unclear. However, the existence of such stable resonances is apparent, considering, for example, the case of Mode 1 being nonlinearly driven by a combination of Modes 2 and 3 subject to $nf_1 = f_2 + f_3$ and having high energy, stiffness and modal mass, such that the backaction of Mode 1 on them is negligible. Perhaps the simplest possible example of such interaction is encountered by subjecting the Mode 2 of our system to a constant linear drive. Initializing Mode 1 with sufficient energy to lock into a PTS leads to a system locked in a period-tripled symmetry-broken steady state.

References

[1] D. Antonio et al. "Frequency stabilization in nonlinear micromechanical oscillators" Nat Commun **3**, 806 (2012).

[2] C. Chen et al. "Direct observation of coherent energy transfer in nonlinear micromechanical oscillator" Nat Commun **8**, 15523 (2017).

[3] J. Guttinger et al. "Energy dependent path of dissipation in nanomechanical resonators" Nature Nanotech **12**, 631-636 (2017).

[4] J. Gosner et. al. "Relaxation dynamics and dissipative phase transition in quantum oscillators with period tripling" Phys. Rev. B **101**, 054501 (2020).

[5] Y. Zhang et al. "Time-translation-symmetry breaking in a driven oscillator: From the quantum coherent to the incoherent regime" Phys. Rev. A **96**, 052124 (2017)

[6] O. Shoshani et al. "Anomalous decay of nanomechanical modes going through nonlinear resonance" Sci Rep **7**, 18091 (2017)
